# Radiomics reveals the biological basis for non-small cell lung cancer prognostic stratification by reflecting tumor immune microenvironment heterogeneity

**DOI:** 10.3389/fimmu.2025.1708692

**Published:** 2025-11-10

**Authors:** Qing Huang, Nie Xu, Jun Yin, Peng Diao, Tianpeng Xie, Ke Xu

**Affiliations:** 1 Department of Radiotherapy, Sichuan Cancer Hospital & Institute, Sichuan Cancer Center, School of Medicine, University of Electronic Science and Technology of China, Chengdu, Sichuan, China; 2 Department of Oncology, Chengdu First Peoples’ Hospital, Integrated Traditional Chinese Medicine (TCM) & Western Medicine Hospital, Chengdu University of Traditional Chinese Medicine, Chengdu, Sichuan, China; 3 Department of Thoracic Surgery, Sichuan Cancer Hospital & Institute, Sichuan Cancer Center, School of Medicine, University of Electronic Science and Technology of China, Chengdu, Sichuan, China

**Keywords:** non-small cell lung cancer, radiomics, prognostic stratification, tumor immune microenvironment, biological basis

## Abstract

**Background:**

Current radiomic non-small cell lung cancer prognostic models predominantly depend on statistical correlations, lacking robust biological validation. This study integrates multi-omics data to develop a preoperative computed tomography (CT) radiomics model, systematically elucidating its biological links to tumor molecular heterogeneity, immune microenvironment, and clinicopathological phenotypes, advancing clinical translation of radiomics.

**Methods:**

This retrospective study analyzed 334 surgically resected stage I-IIIA NSCLC patients. Radiomic features were extracted from preoperative contrast-enhanced CT images. LASSO-Cox regression developed the Rad-score. Cross-cohort validation applied fixed feature thresholds. Integrated gene set enrichment analysis, differential gene expression, and immune microenvironment analyses revealed biological disparities between radiomics risk-stratified groups. Integrated clinicopathological data explored radiomics risk stratification and clinical phenotype associations, constructing a tripartite cross-scale explanatory framework of radiomics-genomics-clinical phenotypes.

**Results:**

The Rad-score demonstrated robust prognostic stratification capacity across the training, internal validation, and external validation cohorts. Gene set enrichment analysis revealed significant enrichment of tumor invasion and proliferation-related pathways—including hypoxia, TNFA-NF-κB signaling, inflammatory response, and angiogenesis—in the high-risk group. Differential gene analysis further identified marked disparities in cell cycle regulation, DNA repair, and platinum resistance between risk groups. Immune microenvironment profiling showed significantly reduced immune scores and decreased proportions of naive B cells in high-risk patients, indicating impaired immune activity. At the macro level, the high-risk group exhibited stronger inflammatory responses, more aggressive clinicopathological phenotypes, and poorer nutritional status, mutually validated by micro-genomic characteristics.

**Conclusion:**

This study demonstrates that radiomics can non-invasively reveal tumor molecular heterogeneity and immune microenvironment characteristics, elucidating direct associations between imaging features and tumor biological behavior. These findings provide a critical theoretical foundation for the clinical translation of radiomics.

## 1 Introduction

Lung cancer remains the malignancy with the highest incidence and mortality rates worldwide, with non-small cell lung cancer (NSCLC) accounting for 85% of all cases (1, 2). Despite significant improvements in patient survival rates achieved through advancements in curative surgical resection and postoperative adjuvant therapies (3, 4), exemplified by early screening and standardized surgical techniques, current prognostic evaluation systems dependent on static parameters such as pathological staging and histological subtypes remain inadequate in explaining the heterogeneity of clinical outcomes among patients with comparable clinicopathological profiles (5–7). Despite achieving complete tumor resection and adhering to standardized adjuvant therapy protocols, 20-25% of NSCLC patients ultimately develop disease recurrence or distant metastases, demanding intensified therapeutic interventions (8, 9). This persistent clinical challenge underscores that refined risk stratification has emerged as a critical imperative for optimizing therapeutic decision-making.

Tumors exhibit spatial and temporal heterogeneity at multiple biological levels, including genetic, proteomic, cellular, tissue, and organ levels, which contribute significantly to tumor invasiveness and therapeutic resistance (10, 11). Tumor heterogeneity refers to the inherent diversity within a tumor, encompassing genetic, phenotypic, and microenvironmental variations (12). This heterogeneity is observed not only between different patients but also within distinct regions of the same tumor in an individual patient. Such diversity allows cancer cells to exhibit variable characteristics, such as differential growth rates, metabolic profiles, and drug resistance, thereby enabling tumors to evade therapeutic pressures and facilitating tumor progression and metastasis (13). Additionally, tumor heterogeneity extends to the immune microenvironment, wherein variations in immune cell infiltration and immune evasion mechanisms further compromise immune surveillance, thereby influencing patient prognosis (14). Extensive research has established that tumor heterogeneity is closely associated with patient outcomes, particularly with respect to genomic and immune microenvironmental factors (15–17).

Medical imaging offers a non-invasive approach for the dynamic observation of tumors and their microenvironment, facilitating a comprehensive evaluation of tumor heterogeneity. Recent advancements in imaging technologies, including innovations in medical imaging equipment, contrast agents, and image analysis techniques, have led to the standardization of image acquisition, driving the progression of medical imaging toward a more quantitative paradigm. Radiomics involves the extraction of quantitative features from medical image data, which, when coupled with machine learning algorithms, can elucidate the biological characteristics of tumors and their relationship with patient prognosis (18). Since its inception, radiomics has achieved substantial progress in various domains, including early cancer detection, treatment monitoring, and prognostic assessment (9, 19–21). Radiomic features are primarily characterized by mathematical descriptions of attributes such as grayscale distribution, texture patterns, and shape features within medical images, reflecting the macroscopic aspects of the images. However, the specific relationships between these quantitative features and the molecular biological characteristics of tumors (such as gene expression, tumor microenvironment) as well as cellular pathological changes remain inadequately understood. Consequently, the absence of a robust biological foundation in radiomic features limits their capacity to elucidate the causal relationships underlying tumor behaviors, such as invasiveness, growth dynamics, treatment sensitivity, and prognosis, thereby hindering their broader clinical application.

To bridge this translational gap, our study pioneers a multidimensional analysis integrating radiomic profiles, genomic landscapes, and clinical-pathological parameters. We systematically interrogate radiomic signatures through a dual-scale framework encompassing molecular-level biological mechanisms (microscopic) and tumor-host interface dynamics (macroscopic). This innovative paradigm aims to elucidate the biological underpinnings of radiomic signatures while enhancing their translational utility through mechanistic correlations with tumor evolution patterns and therapeutic vulnerabilities.

## 2 Methods

### 2.1 Patients

**Figure 1:**
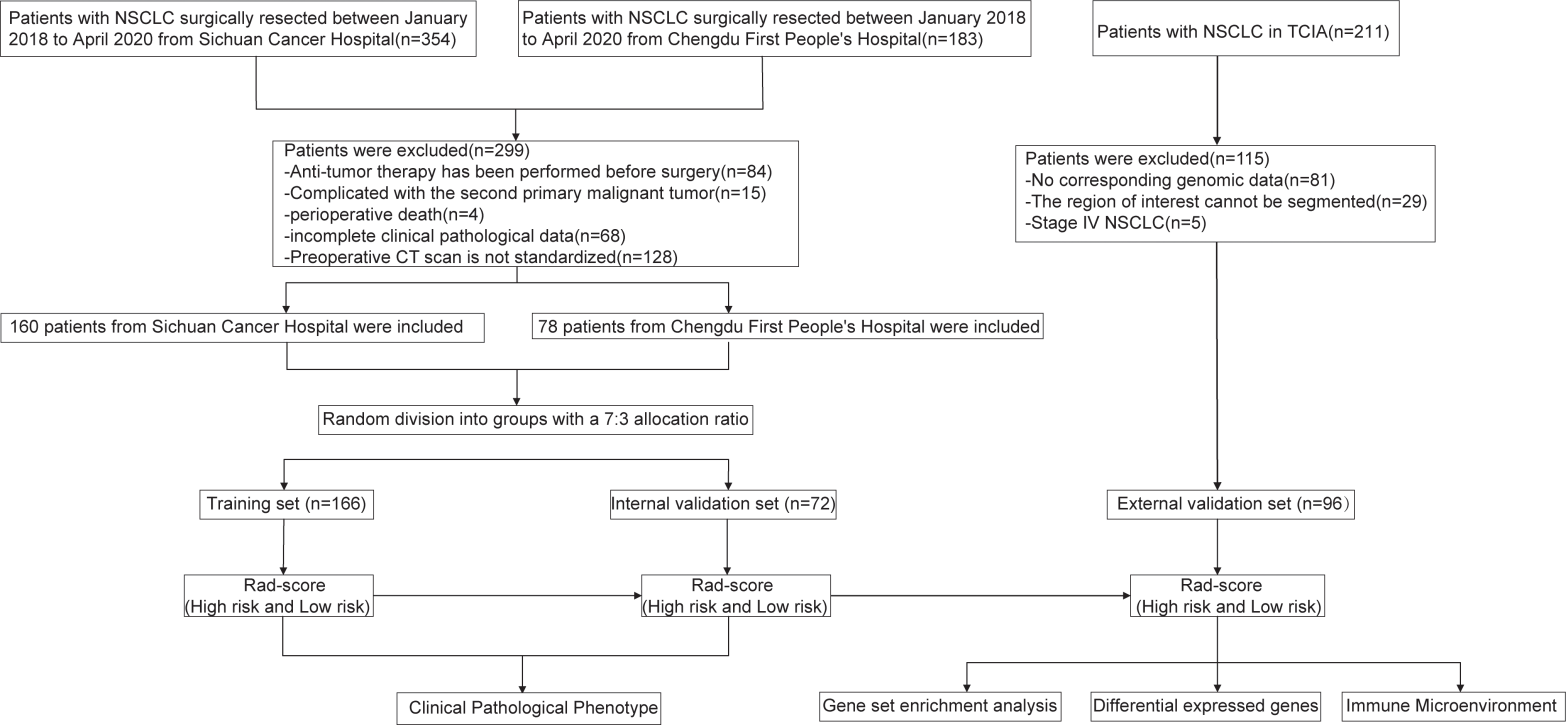
The overview of this study workflow.

The patient enrollment process and the study flowchart are presented in **Figure 1**. The study population consisted of a training set, an internal validation set, and an external validation set. NSCLC patients who underwent radical surgical resection at Sichuan Cancer Hospital and Chengdu First People’s Hospital between January 1, 2018, and April 1, 2020, were included. The inclusion criteria were: (1) post-operative histopathological confirmation of NSCLC; (2) a chest-enhanced computed tomography (CT) scan within one month prior to surgery; (3) preoperative imaging confirming the absence of distant metastasis; (IV) availability of complete follow-up data. Exclusion criteria included: (1) prior chemotherapy, radiotherapy, or other anti-tumor treatments before surgery; (2) presence of a second primary malignancy or other types of malignant tumors; (3) perioperative mortality; (4) incomplete clinical-pathological data. A total of 354 patients from Sichuan Cancer Hospital and 183 patients from Chengdu First People’s Hospital underwent radical resection for NSCLC between January 1, 2018, and April 1, 2020. After applying the inclusion and exclusion criteria, 238 patients were ultimately enrolled (160 from Sichuan Cancer Hospital and 78 from Chengdu First People’s Hospital). All patients were randomly assigned to either the training set (166 patients) or the internal validation set (72 patients) in a 7:3 ratio. The external validation set was derived from the NSCLC Radiogenomics database within The Cancer Imaging Archive (TCIA) (https://www.cancerimagingarchive.net/), initially including 211 patients (22). After excluding those without genomic data or with CT images that could not delineate the lesions, 96 patients were ultimately included. This study was conducted in accordance with the Helsinki Declaration (revised in 2013) and was approved by Ethics Committee for Medical Research and New Medical Technology of Sichuan Cancer Hospital (Approval No. SCCHEC-2022-118). Informed consent was waived due to the retrospective study design.

### 2.2 Study endpoints and follow-up

The primary endpoint of this study is disease-free survival (DFS), defined as the interval from the date of surgery to the first occurrence of tumor recurrence, metastasis, or death from any cause. The secondary endpoint is overall survival (OS), defined as the interval from the date of surgery to either death or the last follow-up. All enrolled patients underwent preoperative imaging, including enhanced chest CT, upper abdominal enhanced CT or ultrasound, and enhanced head magnetic resonance imaging (MRI) or CT, to determine the disease stage. Postoperatively, patients were monitored according to a standardized follow-up protocol: follow-up every three months during the first two years, every six months between two and five years, and annually thereafter. The follow-up assessments included unenhanced chest CT, abdominal CT or ultrasound, complete blood counts, and liver and kidney function tests. If suspicious lesions or clinical symptoms were identified, patients could undergo further diagnostic tests, such as enhanced CT, MRI, or positron emission tomography/CT (PET/CT), as necessary. The follow-up concluded on December 31, 2023. Data for the external validation set were sourced from TCIA.

### 2.3 CT image acquisition

Preoperative chest contrast-enhanced CT imaging was conducted on all patients using a Siemens 64-slice spiral CT scanner. The scan range extended from the apex to the base of the lungs. Initially, a routine non-contrast scan was performed, followed by a contrast-enhanced scan. For the enhanced scan, iodixanol (100 ml, containing 65.2 g of iodine) was administered via the antecubital vein using a high-pressure injector, at a dose of 1.5 mg/kg, followed by 20 ml of saline at an injection rate of 2.5 ml/s. Arterial-phase and venous-phase imaging were acquired at 25- and 60-seconds post-injection, respectively. The scan parameters were as follows: tube voltage 120 kV, tube current 250 mAs, slice thickness of 5 mm with a 5 mm slice interval, and a matrix size of 512×512. The CT images for the external validation set were obtained from TCIA.

### 2.4 Image segmentation and feature extraction

A radiation oncologist with over 10 years of experience in lung cancer radiotherapy manually delineated the tumor boundaries and defined the regions of interest (ROI) on preoperative contrast-enhanced CT axial images using 3D Slicer 5.6.2 software. The tumor was fully covered during ROI delineation, with regions corresponding to large blood vessels, bronchi, and surrounding healthy lung tissue excluded. Subsequently, image preprocessing and radiomic feature extraction were performed using the PyRadiomics 3.0.1 package. A total of 851 radiomic features were extracted for each patient, including: (1) 14 shape features; (2) 18 first-order features; (3) 24 Gray-Level Co-occurrence Matrix​ (GLCM) features; (4) 16 ​Gray-Level Run-Length Matrix​ (GLRLM) features; (5) 16 Gray-Level Size Zone Matrix (GLSZM) features; (6) 5 Neighboring Gray Tone Difference Matrix (NGTDM) features; (7) 14 Gray-Level Dependence Matrix​ (GLDM) features; and (8) 744 wavelet features. All feature parameters were Z-score normalized using data from the training set. To assess intra-observer reproducibility, the radiation oncologist repeated the ROI delineation and feature extraction for 50 randomly selected patients within one week. For inter-observer reproducibility, another senior radiation oncologist with extensive experience in lung cancer radiotherapy performed the same tasks on the same set of 50 patients. The intra-class correlation coefficient (ICC) was calculated through consistency testing to evaluate feature stability.

### 2.5 Feature selection and radiomic score construction

This study employs a multi-step algorithmic approach for dimensionality reduction of high-dimensional data. The process is outlined as follows: (1) Selection of feature parameters exhibiting high stability, as evidenced by both intra- and inter-observer consistency tests, with ICC greater than 0.90. (2) Integration of the maximal relevance and minimal redundancy (MRMR) and random survival forest (RSF) algorithms to identify the top 20 radiomic features based on their scores or importance rankings. (3) Further feature selection is performed using the Least Absolute Shrinkage and Selection Operator (LASSO)-Cox method, with 10-fold cross-validation used to determine the optimal weight parameter λ, resulting in the formulation of a linear combination for the radiomics score (Rad-score).

### 2.6 Prognostic significance of Rad-score

The association between the Rad-score and DFS was evaluated in both the training and validation cohorts. The predictive performance of the Rad-score was assessed through time-dependent receiver operating characteristic (ROC) curves, with the optimal cutoff value determined. Patients were then stratified into high risk and low risk groups based on this cutoff. The prognostic value of the Rad-score was further validated externally using the NSCLC Radiogenomics cohort from TCIA. The same radiomic scoring formula and cutoff value were applied to categorize patients in the external validation set into high risk and low risk groups. Finally, survival differences between the risk groups were analyzed using Kaplan-Meier survival curves.

### 2.7 Construction and validation of the prognostic model

Clinical parameters were incorporated into both univariate and multivariate Cox regression models to identify independent prognostic factors and develop a clinical prognostic model. A combined prognostic model was subsequently established by integrating clinical parameters with the Rad-score. This model was first assessed in the training cohort and later validated in both internal and external validation cohorts.

### 2.8 Gene enrichment analysis of risk groups based on Rad-score

To investigate the biological significance of the Rad-score, this study included 96 NSCLC patients with RNA-seq data from the NSCLC Radiogenomics cohort in the NCBI (https://www.ncbi.nlm.nih.gov/). These patients were stratified into three groups based on the Rad-score: the top 36 were classified as the high-risk group, the middle 32 as the normal-risk group, and the bottom 32 as the low-risk group. RNA-seq data for these patients were subsequently retrieved from the GSE103584 dataset in the GEO database. Gene set enrichment analysis (GSEA) was conducted using the MSigDB Hallmark gene set to identify biological pathways associated with the high-risk and low-risk groups. A false discovery rate (FDR) of less than 0.25 was deemed statistically significant.

### 2.9 Differential expressed genes of risk groups based on Rad-score

Differentially expressed genes between the high-risk and low-risk groups were identified using the “limma” and “edgeR” packages in R, applying selection criteria of Log2|FC| > 0.1 and *P* < 0.05. Gene Ontology (GO) enrichment analysis and Kyoto Encyclopedia of Genes and Genomes (KEGG) pathway analysis were subsequently conducted based on these differentials expressed genes, utilizing the Metascape and “clusterProfiler” packages.

### 2.10 Immune microenvironment of risk groups based on Rad-score

The ESTIMATE algorithm was employed to estimate tumor purity in the high-risk and low-risk patient groups (23). Immune phenotyping scores were derived for each patient (https://tcia.at/tools/toolsMain), and differences in immune phenotypes were assessed using the Kolmogorov-Smirnov test to identify potential variations in immune profiles (24). The cell-type identification by estimating relative subsets of RNA transcripts(CIBERSORT) algorithm from the R package ​immuno-oncology biological research (IOBR) was utilized to quantify the infiltration levels of 22 distinct immune cell types in each sample (25). Chi-square tests were applied to compare the abundance of these immune cell types between the high-risk and low-risk groups, providing insights into the potential immune microenvironmental differences between the two groups.

### 2.11 Correlation of clinical pathological parameters with Rad-score

At the macro level, this study examined the association between Rad-score and clinical pathological parameters in 238 patients with NSCLC. Specifically, inflammatory and nutritional markers, such as white blood cell (WBC) count, neutrophil count, monocyte count, neutrophil-to-lymphocyte ratio (NLR), platelet-to-lymphocyte ratio (PLR), systemic immune-inflammation index (SII), prognostic nutritional index (PNI), and albumin levels, were analyzed. Furthermore, the relationship between Rad-score and pathological phenotypes was assessed, including T-stage, N-stage, tumor differentiation, number of positive lymph nodes, tumor diameter, and Ki67 expression levels.

### 2.12 Statistical analysis

Statistical analyses were conducted using RStudio 4.3.1. The “survminer” R package was employed to determine the optimal cut-off values for continuous variables via the surv_cutpoint function. For skewed data, the interquartile range (IQR) was utilized to summarize the data, and inter-group comparisons were performed using the Mann-Whitney U test. Categorical data were expressed as counts or percentages, with comparisons between groups carried out using the χ² test. The cut-off value for the Rad-score was established through ROC curve analysis, with its discriminatory power assessed by the area under the curve (AUC). Kaplan-Meier survival curves were constructed to estimate survival rates, and the Log-rank test was employed to evaluate differences in survival between groups. Furthermore, the concordance index (C-index) was computed, along with time-dependent ROC curves and decision curve analysis (DCA) to assess the predictive performance of the model. The DeLong test was used to compare AUC differences between models. Nomograms and calibration curves were generated based on the optimal model to evaluate the agreement between predicted and actual outcomes. A *P*-value of <0.05 was considered statistically significant.

## 3 Results

### 3.1 Patient characteristics

In this study, 238 patients with NSCLC who underwent radical surgical resection were enrolled and followed up. The cohort included 165 males and 73 females, with a median age of 61 years (range: 30–82 years) and a median follow-up duration of 50 months. During the follow-up period, 97 patients died, and 121 developed recurrence or metastasis. In the training cohort, the median follow-up duration was 49 months (range: 3–72 months). The DFS rates at 1, 3, and 5 years were 73%, 54%, and 49%, respectively, while the OS rates were 91%, 67%, and 57%, respectively. In the internal validation cohort, the median follow-up duration was 51 months (range: 3–71 months). The 1, 3, and 5-year DFS rates were 81%, 54%, and 45%, respectively, with corresponding OS rates of 92%, 70%, and 59%. No statistically significant differences in survival outcomes were observed between the training and internal validation cohorts (*P >*0.05). Additionally, comparisons of clinicopathological characteristics between the two cohorts revealed no significant differences (*P >*0.05), indicating high comparability between the groups. Detailed results are presented in **Table 1**.

**Table 1:** The baseline characteristics of patients with NSCLC in the training and validation cohorts.

Variables		Total(n = 238)	Training(n = 166)	Validation(n = 72)	*P*
Gender	Male	165(69.3%)	120(50.4%)	45(18.9%)	0.132
	Female	73(30.7%)	46(19.3%)	27(11.4%)	
Age (year)	median [IQR]	61[54-66]	60.5[54-67]	62[56-65]	0.723
Smoking history	Smoker	132(55.5%)	94(39.5%)	38(16.0%)	0.583
	Non-smoker	106(44.5%)	72(30.2%)	34(14.3%)	
Type of surgery	Lobectomy	196(82.3%)	139(58.4%)	57(23.9%)	0.690
	Bilobectomy	23(9.7%)	15(6.3%)	8(3.4%)	
	Pneumonectomy	19(8.0%)	12(5.0%)	7(2.9%)	
Histopathology	Adenocarcinoma	135(56.7%)	89(37.4%)	46(19.3%)	0.135
	Squamous cell	97(40.8%)	74(31.1%)	23(9.7%)	
	Adenosquamous	6(2.5%)	3(1.3%)	3(1.3%)	
T stage	T1-2	184(77.3%)	129(54.2%)	55(23.1%)	0.823
	T3-4	54(22.7%)	37(15.5%)	17(7.1%)	
N stage	N0-1	169(71%)	115(48.3%)	54(22.7%)	0.371
	N2	69(29%)	51(21.4%)	18(7.6%)	
UICC stage	I-II	150(63%)	103(43.3%)	47(19.7%)	0.635
	I	88(37%)	63(26.5%)	25(10.5%)	
Number of lymph node dissection	<16	103(43.3%)	69(29.0%)	34(14.3%)	0.419
	≥16	135(56.7%)	97(40.8%)	38(16.0%)	
Lymph node status	Negative	130(54.6%)	87(36.6%)	43(18.1%)	0.298
	Positive	108(45.4%)	79(33.2%)	29(12.2%)	
Differentiation	Poor	142(59.7%)	103(43.3%)	39(16.4%)	0.255
	Moderate and well	96(40.3%)	63(26.5%)	33(13.9%)	
Radiotherapy	Yes	14(5.9%)	12(5.0%)	2(0.8%)	0.180
	No	224(94.1%)	154(64.7%)	70(29.4%)	
Chemotherapy	Yes	123(51.7%)	75(31.5%)	40(16.8%)	0.141
	No	115(48.3%)	91(38.2%)	32(13.4%)	
Hemoglobin, g/L	median [IQR]	134[122-144]	135[122-144]	130[121-142]	0.418
Albumin, g/L	median [IQR]	43[40-45]	43[40-45]	43[39-45]	0.698
NLR	median [IQR]	2.8[2.0-3.7]	3.0[2.0-3.7]	2.4[1.8-3.4]	0.104
PLR	median [IQR]	122[93-168]	121[93-170]	131[93-167]	0.555
SII	median [IQR]	519[333-809]	540[338-830]	462[284-695]	0.160
PNI	median [IQR]	51[47-54]	51[48-54]	50[47-53]	0.191
GNRI	median [IQR]	107[100-113]	107[101-113]	105[98-113]	0.268
CONUT score	<3	214(89.9%)	151(63.4%)	63(26.5%)	0.415
	≥3	24(10.1%)	15(6.3%)	9(3.8%)	
Recurrence	Yes	121(50.8%)	83(34.9%)	38(16%)	0.694
	No	117(49.2%)	83(34.9%)	34(14.3%)	

IQR, interquartile ranges; UICC, Union for International Cancer Control; NLR, neutrophil-lymphocyte ratio; PLR, platelet-to-lymphocyte ratio; SII, systemic immune-inflammation index; PNI, prognostic nutritional index; GNRI, geriatric nutritional risk index; CONUT, controlling nutritional status score.

### 3.2 Radiomics feature extraction, selection, and Rad-score construction

Radiomic features were extracted from the ROIs for all patients, generating 851 features per ROI. Among these, 452 features demonstrating high reproducibility (ICC > 0.90) were selected for subsequent analysis. Using MRMR scores and RSF importance rankings, the top 20 features were identified, from which 38 relevant features were retained (**Supplementary Figure S1**). Dimensionality reduction was subsequently performed using Least Absolute Shrinkage and Selection Operator (LASSO)-Cox regression, resulting in the selection of 4 key radiomic features. The definitions and implications of these four features are detailed in **Supplementary Table S1**. These features were utilized to develop the Rad-score (**Supplementary Figure S2**).

### 3.3 Prognostic performance of the Rad-Score

**Figure 2:**
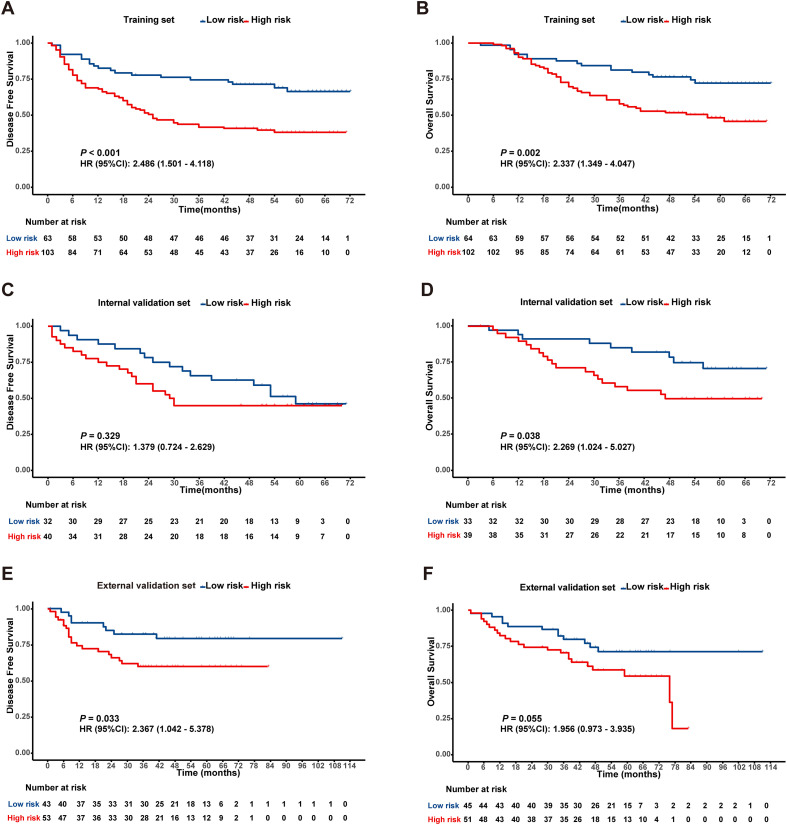
Kaplan-Meier survival analysis according to the best cut off value of the radiomics score in the training set **(A, B)**, internal validation set **(C, D)** and external validation set **(E, F)**.

In the training cohort, ROC analysis for DFS identified a Rad-score cutoff of -0.051 (AUC = 0.643, 95% CI: 0.560-0.727), stratifying patients into risk groups with significant DFS difference (*P* < 0.001, **Figure 2A**). For OS, cutoff -0.043 (AUC = 0.634, 95% CI: 0.549-0.719) yielded significant OS differences in both training (*P* < 0.05, **Figure 2B**) and internal validation cohorts (*P* < 0.05, **Figure 2D**). Although internal validation showed non-significant DFS trend (*P* = 0.329, **Figure 2C**), external validation (TCIA cohort) confirmed significantly shorter DFS and OS in high-risk patients (**Figures 2E, F**).

### 3.4 Univariate and multivariate prognostic analyses in NSCLC

Cox univariate analysis identified tumor diameter, differentiation, T stage, N stage, WBC, PLT, and Rad-score as significant risk factors for DFS (*P* < 0.05). For OS, differentiation, T stage, N stage, WBC, PLT, PLR, SII, PNI, and Rad-score were significant prognostic factors (*P* < 0.05), as detailed in **Table 2**. Cox multivariate analysis confirmed that T stage, N stage, and Rad-score were independent predictors of DFS (*P* < 0.05). Similarly, N stage, PLT, PNI, and Rad-score were identified as independent prognostic factors for OS (*P* < 0.05), as shown in **Table 3**.

**Table 2:** Univariate analysis of prognostic factors in training cohort.

Factors	DFS				OS		
	HR	95% CI	*P*	HR	95% CI	*P*	
Gender	1.020	0.634-1.639	0.935	1.137	0.665-1.947	0.639
Age	1.124	0.729-1.734	0.596	1.428	0.883-2.310	0.146
Smoking history	1.013	0.657-1.562	0.954	1.065	0.660 -1.718	0.795
Tumor diameter	1.602	1.020-2.517	0.041	1.536	0.931- 2.535	0.093
Pathology	1.525	0.977-2.382	0.064	1.508	0.989-2.298	0.056
Differentiation	1.825	1.128-2.953	0.014	1.859	1.084-3.174	0.024
Number of lymph node dissection	1.404	0.913-2.161	0.123	1.117	0.694-1.798	0.649
T stage	1.834	1.146-2.934	0.011	1.750	1.047-2.925	0.033
N stage	3.030	1.958-4.689	<0.001	3.106	1.930-4.997	<0.001
Radiotherapy	1.717	0.827-3.566	0.147	1.353	0.585-3.127	0.480
Chemotherapy	1.540	0.987-2.403	0.057	1.273	0.787-2.059	0.326
WBC	1.727	1.060-2.813	0.028	1.726	1.008-2.956	0.047
NEUT	1.774	0.981-3.209	0.058	1.655	0.869-3.154	0.126
LY	1.419	0.903-2.229	0.129	1.494	0.910-2.454	0.113
MONO	1.353	0.855-2.143	0.197	1.496	0.911-2.455	0.111
PLT	1.776	1.149-2.745	0.010	2.574	1.603-4.132	<0.001
CRP	1.151	0.712-1.862	0.566	1.380	0.826-2.305	0.219
NLR	1.246	0.800-1.939	0.331	1.387	0.849 -2.267	0.192
PLR	1.166	0.758-1.793	0.485	1.876	1.159-3.035	0.010
SII	1.310	0.850-2.018	0.221	1.649	1.019-2.668	0.041
PNI	1.472	0.957-2.264	0.079	1.804	1.123-2.898	0.015
GNRI	1.201	0.686-2.102	0.521	1.597	0.901-2.832	0.109
CONUT	1.248	0.602-2.589	0.551	1.197	0.548-2.616	0.652
Rad-score	2.793	1.593-4.896	<0.001	2.800	1.499-5.231	0.001

DFS, disease-free survival; HR, hazard ratio; CI, confidence interval; WBC, white blood cell; NEUT, Neutrophil; LY, lymphocyte; MONO, monocyte; PLT, platelet; CRP, C-reactive protein; NLR, neutrophil-to-lymphocyte ratio; PLR, platelet-to-lymphocyte ratio; SII, systemic immune-inflammation index; PNI, prognostic nutritional index; GNRI, geriatric nutritional risk index; CONUT, controlling nutritional status score.

**Table 3:** Multivariate analysis of prognostic factors in training cohort.

Factors	DFS			OS		
	HR	95% CI	*P*	HR	95% CI	*P*
Length	1.333	0.672-2.645	0.411	1.798	0.801-4.032	0.155
Differentiation	1.335	0.787-2.262	0.284	1.271	0.691-2.336	0.442
T stage	2.220	1.144-8.704	0.026	1.224	0.688-2.179	0.492
N stage	3.113	1.947-4.978	<0.001	3.164	1.900-5.268	<0.001
WBC	1.746	0.861-3.540	0.122	1.979	0.868-4.511	0.104
PLT	1.503	0.850-2.655	0.161	1.989	1.058-3.739	0.033
PLR	1.239	0.628-2.445	0.537	1.458	0.663-3.208	0.349
SII	1.175	0.610-2.262	0.629	1.426	0.677-3.003	0.350
PNI	1.664	0.979-2.825	0.060	1.842	1.021-3.311	0.042
Rad-score	2.906	1.393-6.060	0.004	3.145	1.391-7.111	0.001

WBC, white blood cell; PLT, platelet; PLR, platelet-to-lymphocyte ratio; SII, systemic immune-inflammation index; PNI, prognostic nutritional index.

### 3.5 Construction and validation of the prognostic model

**Figure 3:**
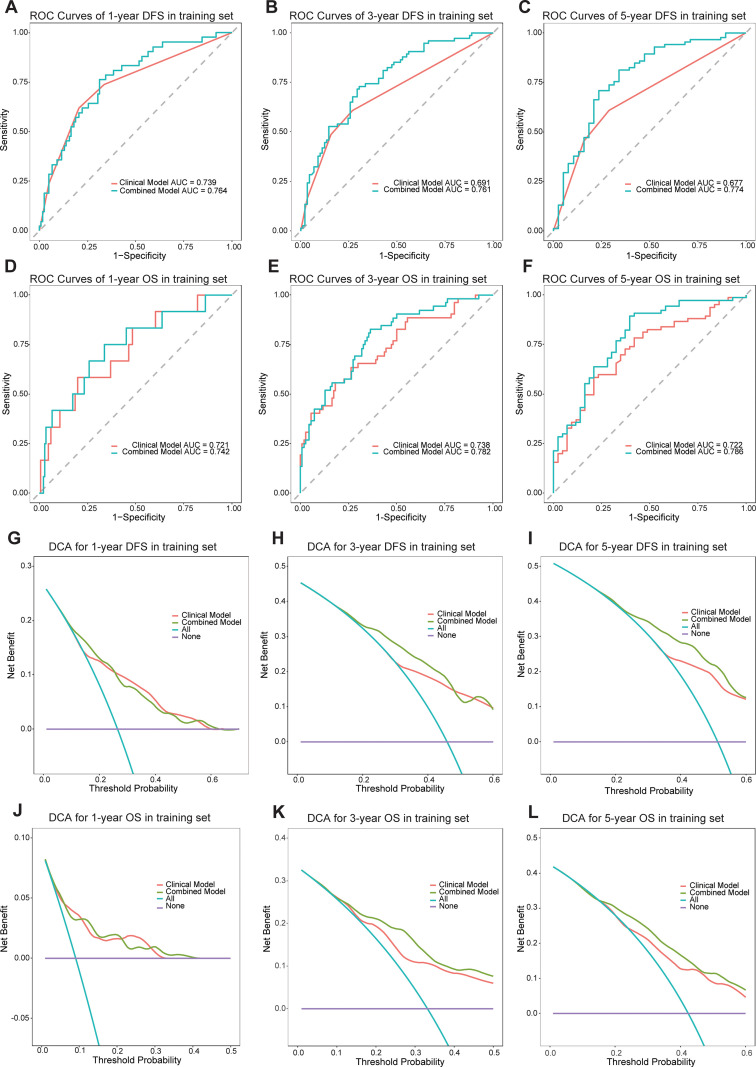
The ROC curves of the models for evaluating the DFS and OS in the training cohorts **(A-F)**. The DCA curves of the models in training cohorts **(G-L)**.

A clinical model was established using significant clinical parameters identified through multivariate analysis. Subsequently, a combined model was developed by incorporating the Rad-score with these clinical parameters. The combined model demonstrated superior predictive efficacy for both DFS and OS compared to the clinical model. In the training cohort, the C-index values for DFS and OS were 0.704 and 0.748, respectively, exceeding the clinical model’s C-index values of 0.655 and 0.684. Similarly, in the validation cohort, the combined model achieved higher C-index values than the clinical model (0.674 vs 0.635 and 0.693 vs 0.664, respectively) (**Supplementary Table S2**). Time-dependent ROC curve analysis indicated that the combined model substantially improved prediction accuracy and diagnostic performance in the training cohort (**Figures 3A–F**). Likewise, in the validation cohort, the combined model outperformed the clinical model in predictive accuracy (**Supplementary Figure S3**). Furthermore, DCA demonstrated that the combined model provided the highest net benefit for predicting both DFS and OS (**Figures 3G–L**, **Supplementary Figure S4**).

### 3.6 Development and validation of the predictive nomogram

**Figure 4:**
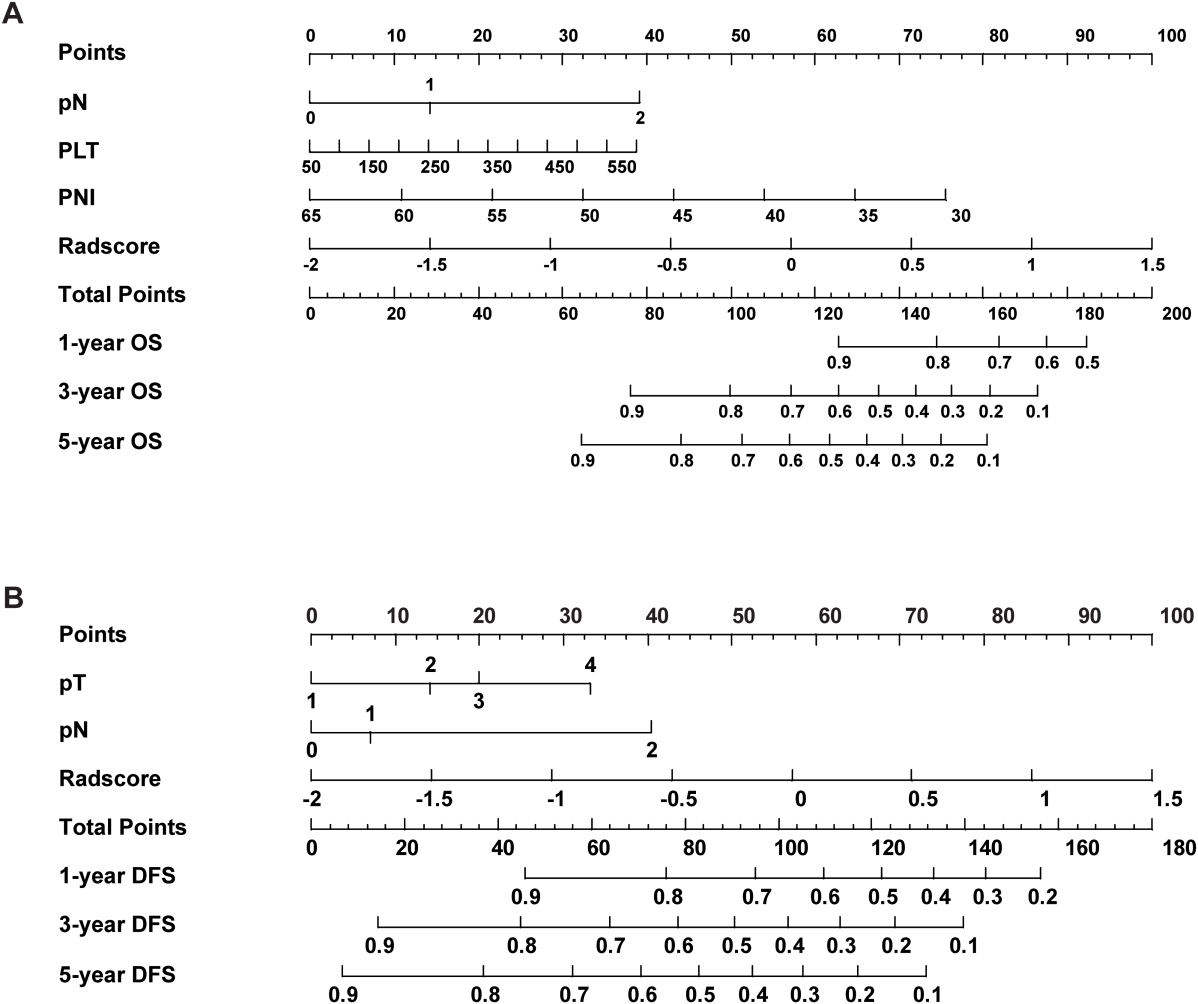
The nomogram combined with the Rad-score and the independent clinical risk factors to predict the risk of DFS **(A)** and OS **(B)**.

A prognostic nomogram for NSCLC was developed based on the combined model by integrating significant prognostic factors. The nomogram serves as an individualized prediction tool for estimating 1-, 3-, and 5-year DFS and OS (**Figure 4**). Incorporating the Rad-score markedly enhanced the model’s predictive accuracy. Calibration curve analysis demonstrated excellent concordance between the nomogram’s predictions and actual observed outcomes for both DFS and OS, indicating the model’s ability to reliably reflect patient survival. These results further confirmed the nomogram’s high reliability in estimating time-specific survival probabilities and underscored its robustness and potential for clinical application (**Supplementary Figure S5**).

### 3.7 Gene set enrichment analysis

**Figure 5:**
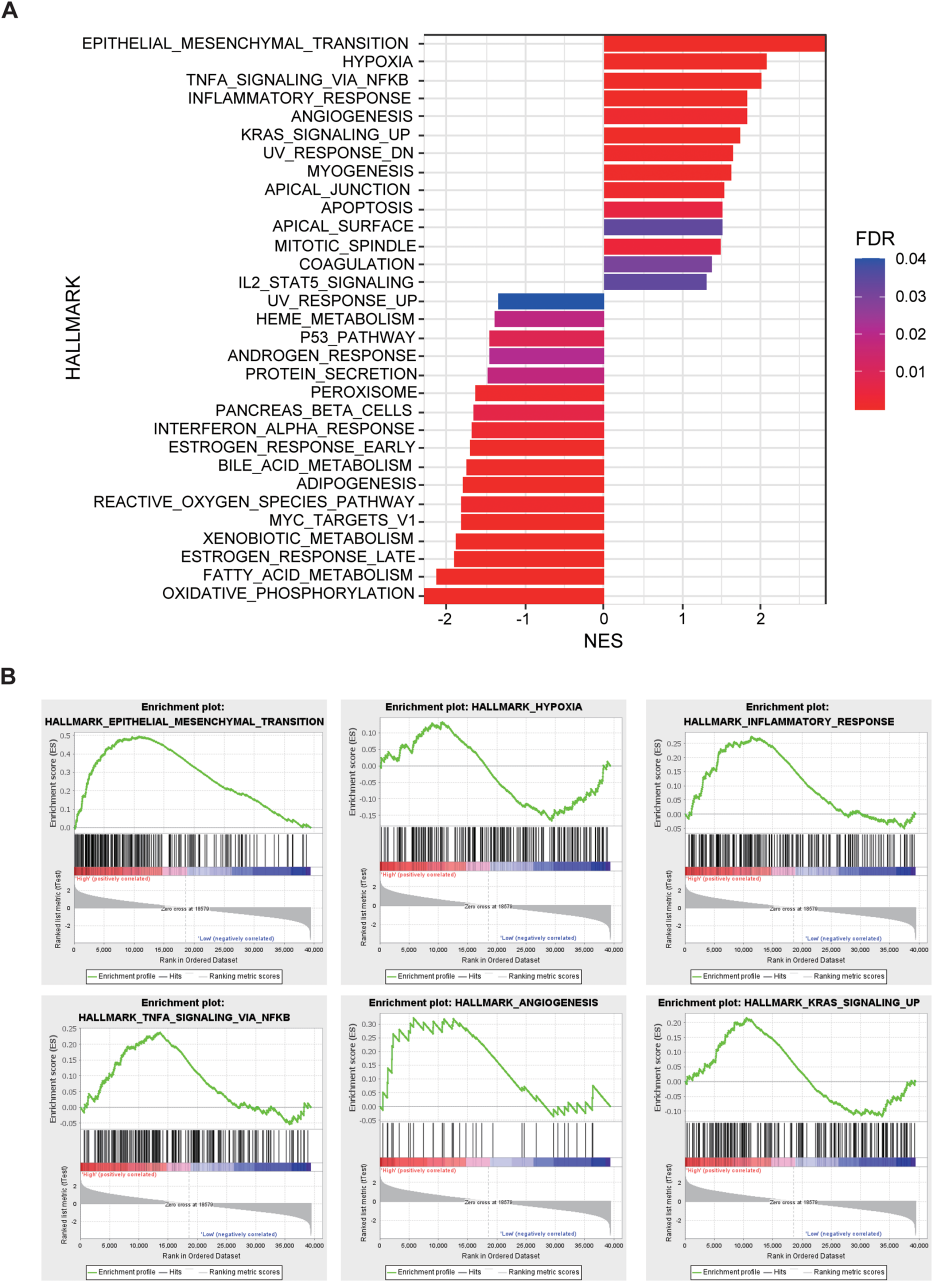
The relationship between Rad-score and gene expression profiles evaluated by gene set enrichment analysis (GSEA). **(A)** GSEA comparing high-risk and low-risk groups stratified by Rad-score. **(B) **Epithelial Mesenchymal Transition, Hypoxia, Inflammatory Response, TNF-α, Angiogenesis, and KRAS signaling up pathways are significantly enriched in the high-risk group.

In the external validation cohort comprising 96 NSCLC patients with RNA-seq data, individuals were stratified into high-risk, normal, and low-risk groups based on the Rad-score. GSEA revealed significant enrichment in the high-risk group for pathways related to epithelial-mesenchymal transition (EMT), hypoxia, TNFA-NF-κB signaling, inflammatory response, angiogenesis, KRAS signaling. Conversely, pathways associated with the P53 signaling pathway, interferon-α response, reactive oxygen species pathway, and oxidative phosphorylation were significantly downregulated in the high-risk group (**Figure 5**). The significant differences in these pathways suggest that tumors in the high-risk group exhibit stronger invasiveness, metastatic potential, immune evasion capability, and drug resistance, along with potential metabolic reprogramming and genomic instability. These biological characteristics may explain the poorer prognosis observed in high-risk group patients.

### 3.8 Differential expressed genes

**Figure 6:**
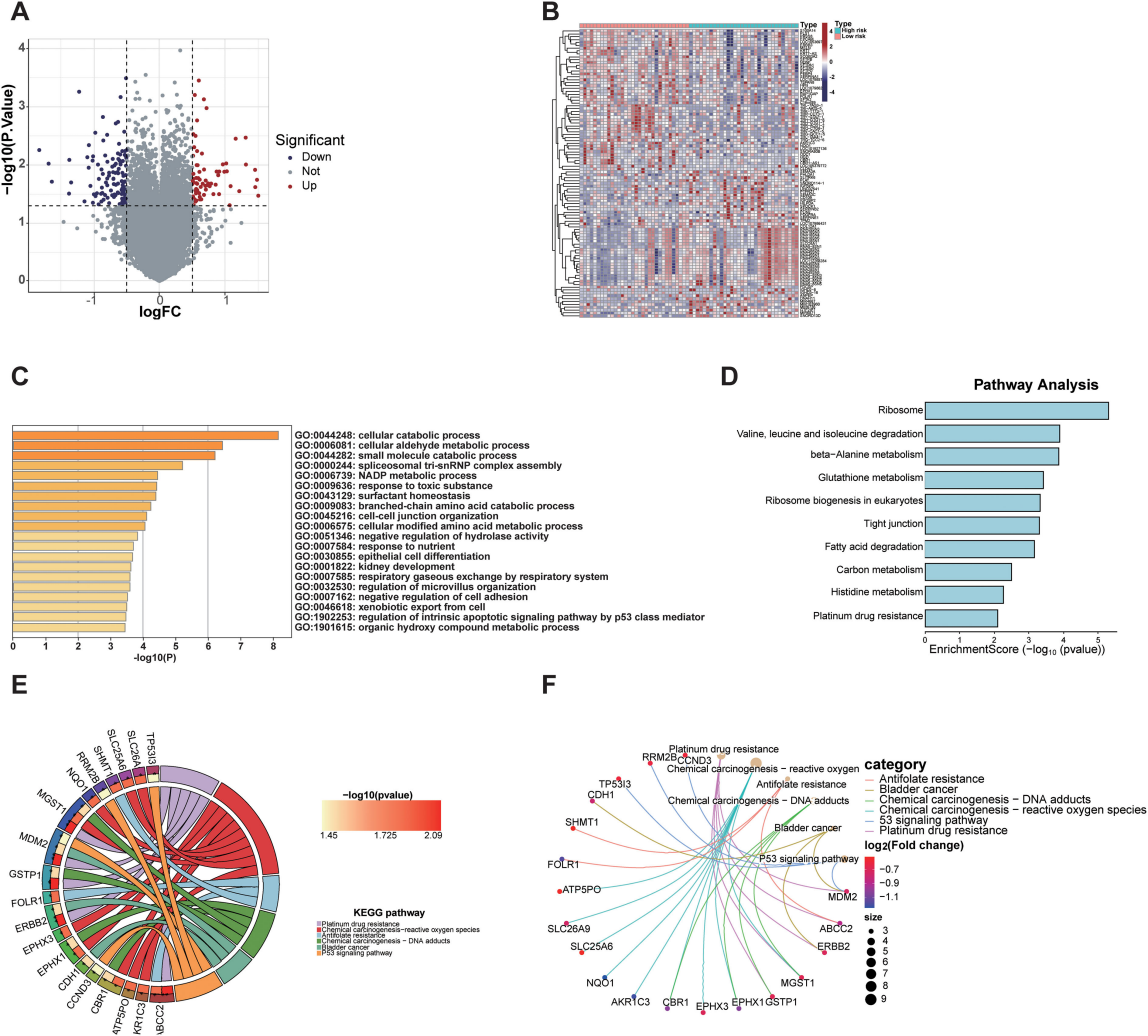
Transcriptomic analysis of differentially expressed genes between radiomics-defined high- and low-risk groups. **(A)** Volcano plot showing significantly upregulated and downregulated genes. **(B)** Heatmap displaying expression patterns of DEGs in high-risk and low-risk groups. **(C)** GO analysis revealed enrichment in cellular catabolic processes, fatty acid metabolism, and extracellular matrix organization. **(D)** KEGG pathway analysis identified enrichment in ribosome biogenesis, glutathione metabolism, and platinum drug resistance. **(E, F)** Gene-pathway network and KEGG chord plot highlighted key pathways associated with tumor progression and drug resistance (26–28).

Differentially expressed gene (DEG) analysis between high-risk and low-risk group identified 71 DEGs significantly upregulated and 116 DEGs significantly downregulated in the high-risk group (**Figure 6A**). These results highlight distinct gene expression profiles distinguishing the high-risk group from the low-risk group (**Figure 6B**). GO enrichment and KEGG pathway analyses of these DEGs revealed significant pathway alterations, including those related to the cell cycle, DNA repair, apoptosis, platinum-based drug resistance, amino acid metabolism, and epithelial cell differentiation (**Figures 6C–F**). These findings reflect distinct biological mechanisms underlying tumor initiation and progression across risk groups and provide a theoretical basis for the biological relevance of radiomics.

### 3.9 Immune microenvironment

**Figure 7:**
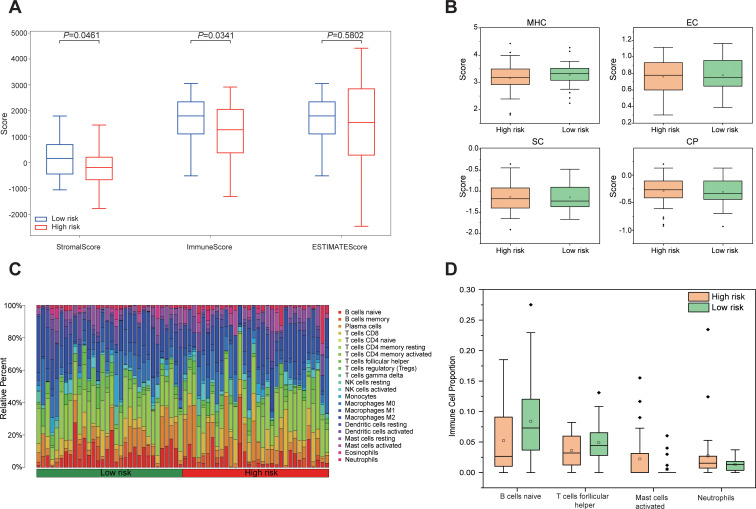
Differences in the immune microenvironment between high-risk and low-risk groups based on Rad-score. **(A)** Boxplots comparing Stromal Score, Immune Score, and ESTIMATE Score between high risk and low risk groups. **(B)** Differences in immunophenotypic characteristics between high- and low-risk groups. **(C)** Stacked bar plot depicting the relative composition of 22 distinct immune cell types between high risk and low risk groups. **(D)** Boxplots comparing four immune cell types with notable differences between high risk and low-risk groups.

Tumor purity was assessed using the ESTIMATE algorithm, revealing no significant difference in the ESTIMATE Score between the high-risk and low-risk group, indicating comparable tumor purity. In contrast, the Immune Score and Stromal score were significantly lower in the high-risk group compared to the low-risk group (*P* < 0.05, **Figure 7A**). Analysis of immune phenotype scores showed that the MHC score was significantly reduced in the high-risk group, while effector cells (EC), suppressor cells (SC), and checkpoints (CP) scores were slightly elevated but did not reach statistical significance (**Figure 7B**). Immune cell composition within the tumor microenvironment was evaluated using CIBERSORTx, estimating the proportions of 22 immune cell types in both groups (**Figure 7C**). The analysis demonstrated a significantly lower proportion of naive B cells in the high-risk group compared to the low-risk group (*P* = 0.043), while follicular helper T cells were marginally reduced but not statistically significant (*P* = 0.089). Additionally, the proportion of activated mast cells was significantly higher in the high-risk group (*P* = 0.02), whereas neutrophils were slightly elevated but did not achieve statistical significance (*P* = 0.087, **Figure 7D**). These results demonstrate that the tumor microenvironment in high-risk group patients exhibits stronger immunosuppressive properties, which may promote tumor progression by impairing anti-tumor immune responses, thereby accounting for their poorer prognosis.

### 3.10 Clinicopathological phenotype

**Figure 8:**
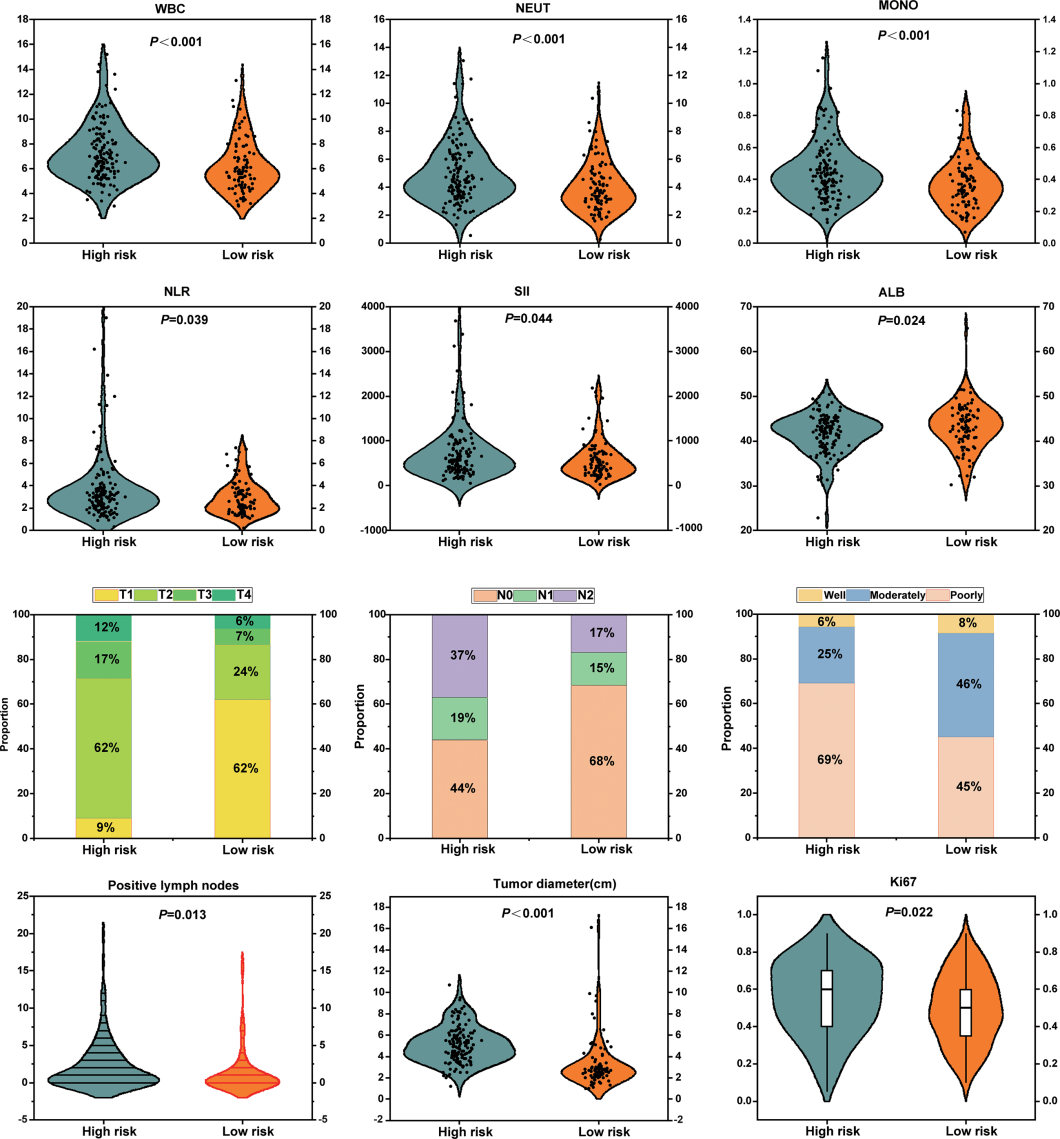
Association analyses between radiomics-based risk stratification with inflammatory-nutritional biomarkers and pathological phenotypes.

At a macroscopic level, the correlation between Rad-score and clinicopathological characteristics, including inflammatory-nutritional indices and pathological staging, was analyzed in 238 patients with NSCLC. The analysis revealed that inflammatory markers, such as WBC, neutrophils (NEUT), monocytes (MOMO), NLR, and SII, were significantly elevated in the high-risk group compared to the low-risk group, while albumin (ALB) levels were significantly reduced. These results suggest that the high-risk group exhibits a heightened inflammatory response and compromised nutritional status (**Figure 8**). Pathologically, the high-risk group demonstrated significantly advanced T and N stages, increased tumor diameter, elevated Ki67 expression, a higher number of positive lymph nodes, and poorer tumor differentiation compared to the low-risk group (**Figure 8**). Enhanced inflammation likely facilitates tumor progression and immune evasion, whereas malnutrition may impair immune function and reduce treatment tolerance. The interplay between these factors may synergistically drive tumor progression and metastasis. This study demonstrates that the multidimensional feature coherence across micro-level (e.g., gene pathway dysregulation, immune microenvironment heterogeneity) and macro-level (e.g., enhanced inflammation, advanced pathological staging, increased tumor invasiveness) aspects reveals the biological mechanisms underlying tumor progression and poor prognosis in the high-risk group, establishing a systematic closed-loop validation from molecular mechanisms to clinical phenotypes.

## 4 Discussion

In the present study, we developed a Rad-score based on preoperative CT imaging features and evaluated its stability and reliability through both internal and external validation. The clinical value of the Rad-score is reflected in its advantages of multidimensional integration, support from biological mechanisms, and its complementary role in clinical decision-making. First, as an independent prognostic factor, the Rad-score significantly enhanced the predictive efficacy of the model when integrated with clinical parameters. Second, its association with key biological pathways such as hypoxia/immune evasion provides an interpretable molecular mechanism basis for the observed prognostic differences. At the clinical application level, the personalized nomogram constructed based on the combined model can assist in identifying high-risk patients missed by traditional TNM staging, thereby optimizing adjuvant treatment decisions.

Radiomic features provide critical insights into the intrinsic heterogeneity of tumors and their underlying biological characteristics, thereby offering more precise tools for prognostic assessment. For example, Dercle et al. demonstrated that baseline CT radiomic features can robustly predict OS in NSCLC patients undergoing immunotherapy or chemotherapy (29).Moreover, a radiomic model developed by Wang et al., incorporating multi-regional features, significantly enhanced the accuracy of postoperative survival risk prediction in patients with stage IA pure solid-type NSCLC31. Similarly, Chen et al. devised a nomogram based on preoperative CT radiomic features and clinical variables, which efficiently predicted lymphovascular invasion and OS in NSCLC patients, showcasing both strong predictive power and clinical applicability (30). In the present study, our multivariate analysis identified the Rad-score as an independent prognostic factor for both DFS and OS, consistent with the findings of Dercle and Wang et al. (29, 31). Although current research primarily centers on the integration of radiomics with clinical features in predictive modeling, it is also well established that inflammation and nutritional status play crucial roles in tumorigenesis, progression, and prognosis (32). Recent studies have increasingly focused on the application of hematological inflammatory and nutritional indices for prognostic evaluation in cancer (33, 34). Our findings further corroborate that the PNI and PLT count are independent risk factors for OS, in line with previous research (35, 36). Notably, this study innovatively integrates radiomic features with inflammatory and nutritional parameters to construct a novel, multidimensional prognostic model. Compared to traditional models based on individual features, this integrated model offers a more comprehensive framework by incorporating the critical roles of inflammation and nutritional status in tumor progression and prognosis, thereby significantly improving the model’s predictive accuracy and clinical utility.

This study provides a comprehensive analysis of the tumor characteristics associated with high- and low-risk groups based on Rad-score at the microscopic level. We observed that the high-risk group is significantly enriched in several signaling pathways related to tumor invasiveness and metastasis, including epithelial-mesenchymal transition (EMT), hypoxia, TNFA-NF-κB signaling, inflammatory response, angiogenesis, KRAS, apoptosis, mitosis, and IL2/STAT5 signaling (37–45). Conversely, tumor-suppressive pathways, such as P53, interferon-α response, reactive oxygen species (ROS), and oxidative phosphorylation (46–49), were markedly downregulated in the high-risk group. These findings provide robust evidence for the biological foundation of radiomics and elucidate the intrinsic relationship between imaging features and tumor molecular mechanisms. Differential gene analysis revealed significant differences between the high- and low-risk groups in various biological processes, including the cell cycle, DNA repair, apoptosis, platinum resistance, amino acid metabolism, and epithelial cell differentiation. These findings suggest that tumors in the high-risk group may exhibit heightened invasiveness, drug resistance, and metabolic adaptability, potentially facilitating accelerated tumor progression and enhancing resistance to therapeutic interventions. Further analysis of the immune microenvironment showed that, although tumor purity was similar between the two groups, the high-risk group exhibited significantly lower immune and stromal scores, indicating reduced immune activity and stromal support. This may contribute to immune escape mechanisms and promote tumor invasiveness and drug resistance. Immune phenotype scoring revealed that the high-risk group had a lower MHC score, reflecting impaired antigen presentation capacity, which further suggests the presence of immune escape mechanisms that could undermine immune surveillance and anti-tumor immune responses. Immune cell composition analysis demonstrated a significantly higher proportion of activated mast cells and a lower proportion of naïve B cells in the high-risk group compared to the low-risk group. These observations suggest a distinct immune cell infiltration pattern in the high-risk group, where an increase in activated mast cells may be linked to inflammatory responses and immune escape mechanisms within the tumor microenvironment (50), while the reduction in B cells may impair immune surveillance and foster tumor progression (51). These findings are consistent with previous studies (23, 52–55). These specific changes in immune cells, combined with the observed decrease in overall immune score and MHC score, collectively characterize an immunosuppressive tumor microenvironment featuring enhanced immunosuppression and a pro-inflammatory state in high-risk tumors. This provides a potential immunological basis for understanding the more aggressive clinicopathological phenotype and poorer prognosis observed in high-risk patient groups. By integrating genomic data, this study identified biological mechanisms and immune microenvironment features significantly associated with Rad-score-based risk stratification. It is crucial to emphasize that these findings (including enriched signaling pathways, differentially expressed genes, and changes in immune cell proportions) represent correlative results from a retrospective observational study, revealing potential biological relationships, and cannot directly prove a causal relationship between radiomic features and molecular/immune alterations.

At the macroscopic phenotypic level, we analyzed the differences between the two groups in terms of inflammatory nutritional indices, pathological staging, and proliferative activity. The findings demonstrated that the high-risk group, as determined by Rad-score, exhibited a pronounced inflammatory response, characterized by elevated levels of WBC, neutrophils, monocytes, NLR, and systemic SII. Additionally, the high-risk group displayed poorer nutritional status, as evidenced by significantly lower albumin levels. Inflammatory responses, through immune system activation and cytokine release, disrupt metabolic processes and exacerbate malnutrition. Inflammation stimulates the release of various pro-inflammatory cytokines, which not only enhance tumor proliferation and invasiveness but also suppress anti-tumor immune responses by secreting immunosuppressive factors, enabling tumor cells to evade immune surveillance and thereby promoting tumor progression (56, 57). Simultaneously, malnutrition leads to deficiencies in proteins and trace elements, impairing immune cell function and hindering the immune system’s ability to recognize and eliminate tumor cells effectively, which further accelerates tumor growth and metastasis (58, 59). The vicious cycle between inflammation and malnutrition not only enhances tumor invasiveness and metabolic adaptability but also reduces treatment tolerance, ultimately contributing to tumor progression. At the pathological phenotypic level, we observed that the high-risk group had more advanced postoperative T and N staging, larger tumor size, higher lymph node involvement, poorer differentiation, and elevated Ki67 expression, underscoring the substantial potential of radiomics in reflecting immune-inflammatory responses within the tumor microenvironment, as well as the processes of tumor proliferation and invasiveness.

While this study has provided valuable insights, several limitations should be acknowledged. First, the sample size restricts the generalizability and predictive accuracy of the clinical prediction model. Although the cases in this study were derived from two centers, the relatively small sample size may undermine the model’s ability to generalize across broader populations. Thus, future research should prioritize increasing the sample size to enhance the model’s robustness and reliability. Second, the external validation cohort (TCIA) exhibits inherent heterogeneity in CT scanning equipment and acquisition parameters, which may compromise the stability of radiomic features. Furthermore, the limited availability of genomic data in the external validation cohort may introduce potential variability and bias. Therefore, larger, independent cohorts with comprehensive genomic data are essential to validate the model’s broader applicability. Lastly, although this study revealed significant differences in molecular pathways and immune infiltration between Rad-score risk stratification groups, these results are inherently observational associations, not proof of causality. Whether these molecular and immune features drive the imaging phenotypes and influence prognostic mechanisms still requires further experimental validation.

## 5 Conclusion

In conclusion, this study provides an innovative examination of the biological foundation of radiomic features, incorporating both the microscopic dimensions of gene pathways and the immune microenvironment, alongside the macroscopic perspectives of inflammatory nutritional indices and clinical pathological phenotypes. This approach offers a novel biological framework for the clinical application of radiomics. The results demonstrate that preoperative CT Rad-score not only predict the prognosis of NSCLC patients undergoing complete surgical resection, but also elucidate the molecular heterogeneity of the tumor and its microenvironment. These findings establish a critical theoretical foundation for the clinical translation of radiomics.

## Data Availability

The datasets presented in this study can be found in online repositories. The names of the repository/repositories and accession number(s) can be found in the article/**supplementary material**.

## References

[1] Han B Zheng R Zeng H Wang S Sun K Chen R . Cancer incidence and mortality in China, 2022. J Natl Cancer Cent. (2024) 4:47–53. doi: 10.1016/j.jncc.2024.01.006, PMID: 39036382 PMC11256708

[2] Cao W Chen H-D Yu Y-W Li N Chen W-Q . Changing profiles of cancer burden worldwide and in China: a secondary analysis of the global cancer statistics 2020. (Engl). (2021) 134:783–91. doi: 10.1097/CM9.0000000000001474, PMID: 33734139 PMC8104205

[3] Kayawake H Okumura N Yamanashi K Otsuki Y Takahashi A Itasaka S . Surgical outcomes and complications of pneumonectomy after induction therapy for non-small cell lung cancer. Gen Thorac Cardiovasc Surg. (2018) 66:658–63. doi: 10.1007/s11748-018-0980-4, PMID: 30083866

[4] Siegel RL Giaquinto AN Jemal A . Cancer statistics, 2024. CA Cancer J Clin. (2024) 74:12–49. doi: 10.3322/caac.21820, PMID: 38230766

[5] Eberhardt WEE Mitchell A Crowley J Kondo H Kim YT Turrisi A International association for study of lung cancer staging and prognostic factors committee, advisory board members, and participating institutions . The IASLC lung cancer staging project: proposals for the revision of the M descriptors in the forthcoming eighth edition of the TNM classification of lung cancer. J Thorac Oncol. (2015) 10:1515–22. doi: 10.1097/JTO.0000000000000673, PMID: 26536193

[6] Kris MG Gaspar LE Chaft JE Kennedy EB Azzoli CG Ellis PM . Adjuvant systemic therapy and adjuvant radiation therapy for stage I to IIIA completely resected non-small-cell lung cancers: american society of clinical oncology/cancer care ontario clinical practice guideline update. J Clin Oncol. (2017) 35:2960–74. doi: 10.1200/JCO.2017.72.4401, PMID: 28437162

[7] Romero-Ventosa EY Blanco-Prieto S González-Piñeiro AL Rodríguez-Berrocal FJ Piñeiro-Corrales G Páez de la Cadena M . Pretreatment levels of the serum biomarkers CEA, CYFRA 21-1, SCC and the soluble EGFR and its ligands EGF, TGF-alpha, HB-EGF in the prediction of outcome in erlotinib treated non-small-cell lung cancer patients. Springerplus. (2015) 4:171. doi: 10.1186/s40064-015-0891-0, PMID: 25918681 PMC4402684

[8] Fick CN Dunne EG Toumbacaris N Tan KS Mastrogiacomo B Park BJ . Late recurrence of completely resected stage I to IIIA lung adenocarcinoma. J Thorac Cardiovasc Surg. (2025) 169:445–453.e3. doi: 10.1016/j.jtcvs.2024.06.026, PMID: 38950771 PMC11682191

[9] Rajaram R Huang Q Li RZ Chandran U Zhang Y Amos TB . Recurrence-free survival in patients with surgically resected non-small cell lung cancer: A systematic literature review and meta-analysis. Chest. (2024) 165:1260–70. doi: 10.1016/j.chest.2023.11.042, PMID: 38065405

[10] Janiszewska M . The microcosmos of intratumor heterogeneity: the space-time of cancer evolution. Oncogene. (2020) 39:2031–9. doi: 10.1038/s41388-019-1127-5, PMID: 31784650 PMC7374939

[11] Dagogo-Jack I Shaw AT . Tumour heterogeneity and resistance to cancer therapies. Nat Rev Clin Oncol. (2018) 15:81–94. doi: 10.1038/nrclinonc.2017.166, PMID: 29115304

[12] Gerlinger M Rowan AJ Horswell S Math M Larkin J Endesfelder D . Intratumor heterogeneity and branched evolution revealed by multiregion sequencing. N Engl J Med. (2012) 366:883–92. doi: 10.1056/NEJMoa1113205, PMID: 22397650 PMC4878653

[13] Hausser J Alon U . Tumour heterogeneity and the evolutionary trade-offs of cancer. Nat Rev Cancer. (2020) 20:247–57. doi: 10.1038/s41568-020-0241-6, PMID: 32094544

[14] Binnewies M Roberts EW Kersten K Chan V Fearon DF Merad M . Understanding the tumor immune microenvironment (TIME) for effective therapy. Nat Med. (2018) 24:541–50. doi: 10.1038/s41591-018-0014-x, PMID: 29686425 PMC5998822

[15] Suresh A Dhanasekaran R . Implications of genetic heterogeneity in hepatocellular cancer. Adv Cancer Res. (2022) 156:103–35. doi: 10.1016/bs.acr.2022.01.007, PMID: 35961697 PMC10321863

[16] Hamidi H Senbabaoglu Y Beig N Roels J Manuel C Guan X . Molecular heterogeneity in urothelial carcinoma and determinants of clinical benefit to PD-L1 blockade. Cancer Cell. (2024) 42:2098–112. doi: 10.1016/j.ccell.2024.10.016, PMID: 39577421

[17] Vitale I Shema E Loi S Galluzzi L . Intratumoral heterogeneity in cancer progression and response to immunotherapy. Nat Med. (2021) 27:212–24. doi: 10.1038/s41591-021-01233-9, PMID: 33574607

[18] Tomaszewski MR Gillies RJ . The biological meaning of radiomic features. Radiology. (2021) 299:E256. doi: 10.1148/radiol.2021219005, PMID: 33900879 PMC8906340

[19] Shin J Seo N Baek S-E Son N-H Lim JS Kim NK . MRI radiomics model predicts pathologic complete response of rectal cancer following chemoradiotherapy. Radiology. (2022) 303:351–8. doi: 10.1148/radiol.211986, PMID: 35133200

[20] Feng Z Li H Liu Q Duan J Zhou W Yu X . CT radiomics to predict macrotrabecular-massive subtype and immune status in hepatocellular carcinoma. Radiology. (2023) 307:e221291. doi: 10.1148/radiol.221291, PMID: 36511807

[21] Hinzpeter R Baumann L Guggenberger R Huellner M Alkadhi H Baessler B . Radiomics for detecting prostate cancer bone metastases invisible in CT: a proof-of-concept study. Eur Radiol. (2022) 32:1823–32. doi: 10.1007/s00330-021-08245-6, PMID: 34559264 PMC8831270

[22] Prior FW Clark K Commean P Freymann J Jaffe C Kirby J . TCIA: An information resource to enable open science. Annu Int Conf IEEE Eng Med Biol Soc. (2013) 2013:1282–5. doi: 10.1109/EMBC.2013.6609742, PMID: 24109929 PMC4257783

[23] Yoshihara K Shahmoradgoli M Martínez E Vegesna R Kim H Torres-Garcia W . Inferring tumour purity and stromal and immune cell admixture from expression data. Nat Commun. (2013) 4:2612. doi: 10.1038/ncomms3612, PMID: 24113773 PMC3826632

[24] Charoentong P Finotello F Angelova M Mayer C Efremova M Rieder D . Pan-cancer immunogenomic analyses reveal genotype-immunophenotype relationships and predictors of response to checkpoint blockade. Cell Rep. (2017) 18:248–62. doi: 10.1016/j.celrep.2016.12.019, PMID: 28052254

[25] Steen CB Liu CL Alizadeh AA Newman AM . Profiling cell type abundance and expression in bulk tissues with CIBERSORTx. Methods Mol Biol. (2020) 2117:135–57. doi: 10.1007/978-1-0716-0301-7_7, PMID: 31960376 PMC7695353

[26] Kanehisa M Furumichi M Sato Y Matsuura Y Ishiguro-Watanabe M . KEGG: biological systems database as a model of the real world. Nucleic Acids Res. (2025) 53:D672–7. doi: 10.1093/nar/gkae909, PMID: 39417505 PMC11701520

[27] Kanehisa M . Toward understanding the origin and evolution of cellular organisms. Protein Sci. (2019) 28:1947–51. doi: 10.1002/pro.3715, PMID: 31441146 PMC6798127

[28] Kanehisa M Goto S . KEGG: kyoto encyclopedia of genes and genomes. Nucleic Acids Res. (2000) 28:27–30. doi: 10.1093/nar/28.1.27, PMID: 10592173 PMC102409

[29] Dercle L Fronheiser M Rizvi NA Hellmann MD Maier S Hayes W . Baseline radiomic signature to estimate overall survival in patients with NSCLC. J Thorac Oncol. (2023) 18:587–98. doi: 10.1016/j.jtho.2022.12.019, PMID: 36646209

[30] Chen Q Shao J Xue T Peng H Li M Duan S . Intratumoral and peritumoral radiomics nomograms for the preoperative prediction of lymphovascular invasion and overall survival in non-small cell lung cancer. Eur Radiol. (2023) 33:947–58. doi: 10.1007/s00330-022-09109-3, PMID: 36064979

[31] Wang T She Y Yang Y Liu X Chen S Zhong Y . Radiomics for survival risk stratification of clinical and pathologic stage IA pure-solid non-small cell lung cancer. Radiology. (2022) 302:425–34. doi: 10.1148/radiol.2021210109, PMID: 34726531

[32] Brunner JS Finley LWS . Metabolic determinants of tumour initiation. Nat Rev Endocrinol. (2023) 19:134–50. doi: 10.1038/s41574-022-00773-5, PMID: 36446897 PMC9936806

[33] Matsui R Rifu K Watanabe J Inaki N Fukunaga T . Impact of malnutrition as defined by the GLIM criteria on treatment outcomes in patients with cancer: A systematic review and meta-analysis. Clin Nutr. (2023) 42:615–24. doi: 10.1016/j.clnu.2023.02.019, PMID: 36931162

[34] Chen N Yu Y Shen W Xu X Fan Y . Nutritional status as prognostic factor of advanced oesophageal cancer patients treated with immune checkpoint inhibitors. Clin Nutr. (2024) 43:142–53. doi: 10.1016/j.clnu.2023.11.030, PMID: 38043419

[35] Park S Ahn HJ Yang M Kim JA Kim JK Park SJ . The prognostic nutritional index and postoperative complications after curative lung cancer resection: A retrospective cohort study. J Thorac Cardiovasc Surg. (2020) 160:276–285.e1. doi: 10.1016/j.jtcvs.2019.10.105, PMID: 31859072

[36] Sandfeld-Paulsen B Aggerholm-Pedersen N Winther-Larsen A . Pretreatment platelet count is a prognostic marker in lung cancer: A danish registry-based cohort study. Clin Lung Cancer. (2023) 24:175–83. doi: 10.1016/j.cllc.2022.12.012, PMID: 36646586

[37] Huang Y Hong W Wei X . The molecular mechanisms and therapeutic strategies of EMT in tumor progression and metastasis. J Hematol Oncol. (2022) 15:129. doi: 10.1186/s13045-022-01347-8, PMID: 36076302 PMC9461252

[38] Chen Z Han F Du Y Shi H Zhou W . Hypoxic microenvironment in cancer: molecular mechanisms and therapeutic interventions. Signal Transduct Targ Ther. (2023) 8:70. doi: 10.1038/s41392-023-01332-8, PMID: 36797231 PMC9935926

[39] Ren X Chen C Luo Y Liu M Li Y Zheng S . lncRNA-PLACT1 sustains activation of NF-κB pathway through a positive feedback loop with IκBα/E2F1 axis in pancreatic cancer. Mol Cancer. (2020) 19:35. doi: 10.1186/s12943-020-01153-1, PMID: 32085715 PMC7033942

[40] Zhao H Wu L Yan G Chen Y Zhou M Wu Y . Inflammation and tumor progression: signaling pathways and targeted intervention. Signal Transduct Targ Ther. (2021) 6:263. doi: 10.1038/s41392-021-00658-5, PMID: 34248142 PMC8273155

[41] Liu ZL Chen HH Zheng LL Sun LP Shi L . Angiogenic signaling pathways and anti-angiogenic therapy for cancer. Signal Transduct Targ Ther. (2023) 8:198. doi: 10.1038/s41392-023-01460-1, PMID: 37169756 PMC10175505

[42] Ma Q Zhang W Wu K Shi L . The roles of KRAS in cancer metabolism, tumor microenvironment and clinical therapy. Mol Cancer. (2025) 24:14. doi: 10.1186/s12943-024-02218-1, PMID: 39806421 PMC11727292

[43] Gourisankar S Krokhotin A Ji W Liu X Chang C-Y Kim SH . Rewiring cancer drivers to activate apoptosis. Nature. (2023) 620:417–25. doi: 10.1038/s41586-023-06348-2, PMID: 37495688 PMC10749586

[44] Kenny TC Craig AJ Villanueva A Germain D . Mitohormesis primes tumor invasion and metastasis. Cell Rep. (2019) 27:2292–2303.e6. doi: 10.1016/j.celrep.2019.04.095, PMID: 31116976 PMC6579120

[45] Lin Y Pu S Wang J Wan Y Wu Z Guo Y . Pancreatic STAT5 activation promotes KrasG12D-induced and inflammation-induced acinar-to-ductal metaplasia and pancreatic cancer. Gut. (2024) 73:1831–43. doi: 10.1136/gutjnl-2024-332225, PMID: 38955401 PMC11503187

[46] Song B Yang P Zhang S . Cell fate regulation governed by p53: Friends or reversible foes in cancer therapy. Cancer Commun (Lond). (2024) 44:297–360. doi: 10.1002/cac2.12520, PMID: 38311377 PMC10958678

[47] Ivashkiv LB . IFNγ: signalling, epigenetics and roles in immunity, metabolism, disease and cancer immunotherapy. Nat Rev Immunol. (2018) 18:545–58. doi: 10.1038/s41577-018-0029-z, PMID: 29921905 PMC6340644

[48] Glorieux C Liu S Trachootham D Huang P . Targeting ROS in cancer: rationale and strategies. Nat Rev Drug Discov. (2024) 23:583–606. doi: 10.1038/s41573-024-00979-4, PMID: 38982305

[49] Wu C Liu Y Liu W Zou T Lu S Zhu C . NNMT-DNMT1 axis is essential for maintaining cancer cell sensitivity to oxidative phosphorylation inhibition. Adv Sci (Weinh). (2022) 10:e2202642. doi: 10.1002/advs.202202642, PMID: 36382559 PMC9811437

[50] Helmink BA Reddy SM Gao J Zhang S Basar R Thakur R . B cells and tertiary lymphoid structures promote immunotherapy response. Nature. (2020) 577:549–55. doi: 10.1038/s41586-019-1922-8, PMID: 31942075 PMC8762581

[51] Wouters MCA Nelson BH . Prognostic significance of tumor-infiltrating B cells and plasma cells in human cancer. Clin Cancer Res. (2018) 24:6125–35. doi: 10.1158/1078-0432.CCR-18-1481, PMID: 30049748

[52] Nie Q Cao H Yang J Liu T Wang B . Integration RNA bulk and single cell RNA sequencing to explore the change of glycolysis-related immune microenvironment and construct prognostic signature in head and neck squamous cell carcinoma. Transl Oncol. (2024) 46:102021. doi: 10.1016/j.tranon.2024.102021, PMID: 38850799 PMC11220558

[53] Huang A Sun Z Hong H Yang Y Chen J Gao Z . Novel hypoxia- and lactate metabolism-related molecular subtyping and prognostic signature for colorectal cancer. J Transl Med. (2024) 22:587. doi: 10.1186/s12967-024-05391-5, PMID: 38902737 PMC11191174

[54] Wang X Xie T Luo J Zhou Z Yu X Guo X . Radiomics predicts the prognosis of patients with locally advanced breast cancer by reflecting the heterogeneity of tumor cells and the tumor microenvironment. Breast Cancer Res. (2022) 24:20. doi: 10.1186/s13058-022-01516-0, PMID: 35292076 PMC8922933

[55] Lv Y Tian W Teng Y Wang P Zhao Y Li Z . Tumor-infiltrating mast cells stimulate ICOS+ regulatory T cells through an IL-33 and IL-2 axis to promote gastric cancer progression. J Adv Res. (2024) 57:149–62. doi: 10.1016/j.jare.2023.04.013, PMID: 37086778 PMC10918354

[56] De Visser KE Joyce JA . The evolving tumor microenvironment: From cancer initiation to metastatic outgrowth. Cancer Cell. (2023) 41:374–403. doi: 10.1016/j.ccell.2023.02.016, PMID: 36917948

[57] Stone ML Lee J Lee JW Coho H Tariveranmoshabad M Wattenberg MM . Hepatocytes coordinate immune evasion in cancer via release of serum amyloid A proteins. Nat Immunol. (2024) 25:755–63. doi: 10.1038/s41590-024-01820-1, PMID: 38641718 PMC11186515

[58] Wang Z Lu Z Lin S Xia J Zhong Z Xie Z . Leucine-tRNA-synthase-2-expressing B cells contribute to colorectal cancer immunoevasion. Immunity. (2022) 55:1067–1081.e8. doi: 10.1016/j.immuni.2022.04.017, PMID: 35659337

[59] Guo C You Z Shi H Sun Y Du X Palacios G . SLC38A2 and glutamine signalling in cDC1s dictate anti-tumour immunity. Nature. (2023) 620:200–8. doi: 10.1038/s41586-023-06299-8, PMID: 37407815 PMC10396969

