# Correction: Radiomics reveals the biological basis for non-small cell lung cancer prognostic stratification by reflecting tumor immune microenvironment heterogeneity

**DOI:** 10.3389/fimmu.2026.1841570

**Published:** 2026-04-09

**Authors:** Qing Huang, Nie Xu, Jun Yin, Peng Diao, Tianpeng Xie, Ke Xu

**Affiliations:** 1 Department of Radiotherapy, Sichuan Cancer Hospital & Institute, Sichuan Cancer Center, School of Medicine, University of Electronic Science and Technology of China, Chengdu, Sichuan, China; 2 Department of Oncology, Chengdu First Peoples’ Hospital, Integrated Traditional Chinese Medicine (TCM) & Western Medicine Hospital, Chengdu University of Traditional Chinese Medicine, Chengdu, Sichuan, China; 3 Department of Thoracic Surgery, Sichuan Cancer Hospital & Institute, Sichuan Cancer Center, School of Medicine, University of Electronic Science and Technology of China, Chengdu, Sichuan, China

**Keywords:** biological basis, non-small cell lung cancer, prognostic stratification, radiomics, tumor immune microenvironment

In the **Methods** section, subsection 2.8 “*Gene enrichment analysis of risk groups based on Rad-score*”, the GEO dataset accession number was incorrectly written. This sentence previously stated:

“RNA-seq data for these patients were subsequently retrieved from the GSE45603 dataset in the GEO database.”

The corrected sentence appears below:

“RNA-seq data for these patients were subsequently retrieved from the GSE103584 dataset in the GEO database.”

The original article has been updated.

