# Supplementary material for: Radiomics reveals the biological basis for non-small cell lung cancer prognostic stratification by reflecting tumor immune microenvironment heterogeneity
Source: Front Immunol. 2025 Nov 10;16:1708692. doi: 10.3389/fimmu.2025.1708692 (PMC12640975; doi:10.3389/fimmu.2025.1708692)
Supplement: Supplementary file 1 [file DataSheet1.docx]

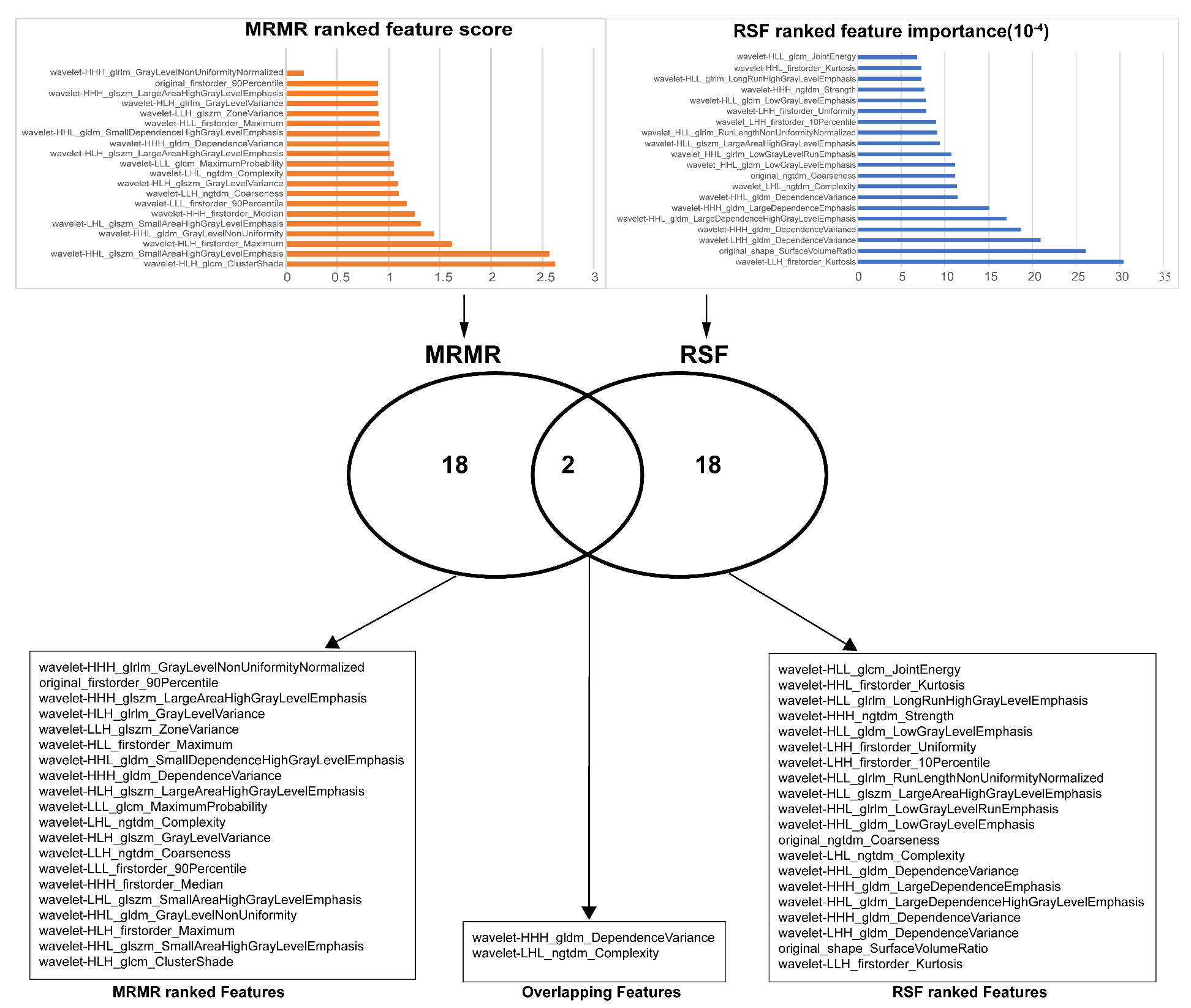


**Figure S1** Top 20 radiomic features ranked by importance using Maximum Relevance Minimum Redundancy and Random Survival Forest algorithms


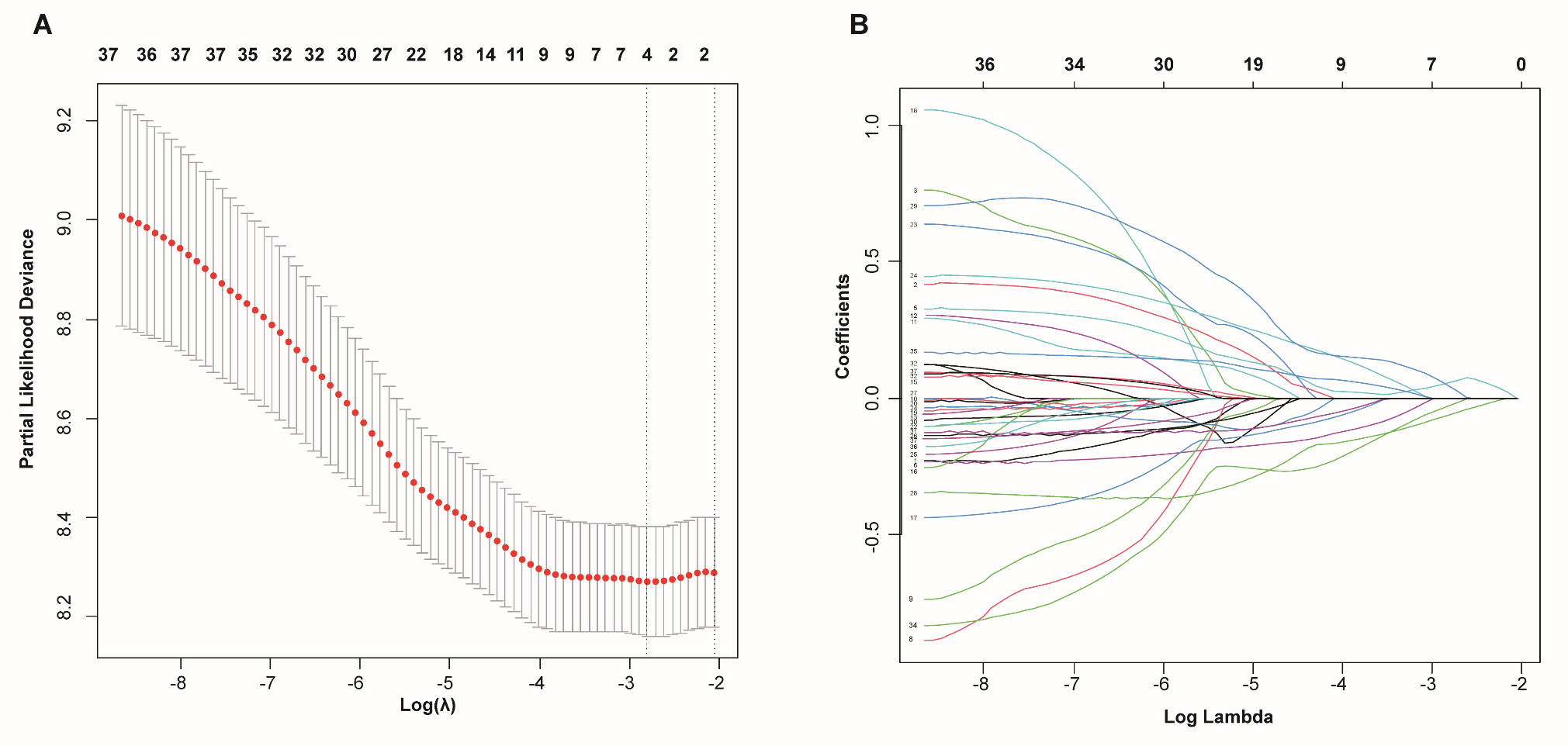


**Figure S2** Using the LASSO-Cox algorithm for further feature selection: **(A)** Based on the minimum likelihood deviation and 10-fold cross-validation method, the optimal weight parameter α=0.061, and log(λ)=−2.797 were selected, resulting in the most efficient feature set. **(B)** The coefficient convergence graph for feature selection using the cross-validation method in the LASSO regression model ultimately identified four radiomics features.

**
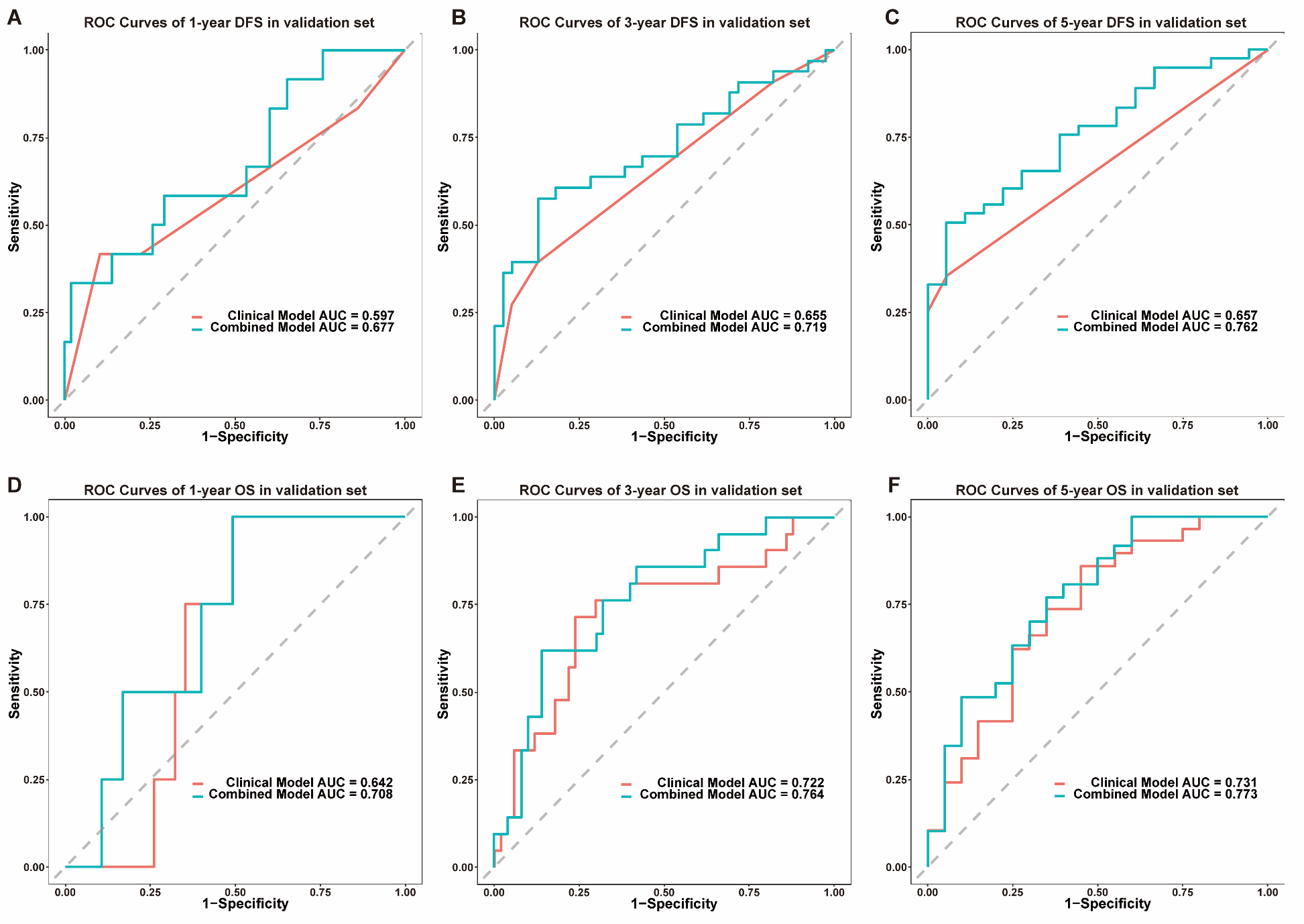
**

**Figure S3** The ROC curves of the models for evaluating the DFS and OS in the validation cohorts. **A-C**, The ROC curves of the comparison between the clinical model and combined model to predict the 1-, 3-, and 5-year DFS. **D-F**, The ROC curves of the comparison between the clinical model and combined model predict the 1-, 3-, and 5-year OS.
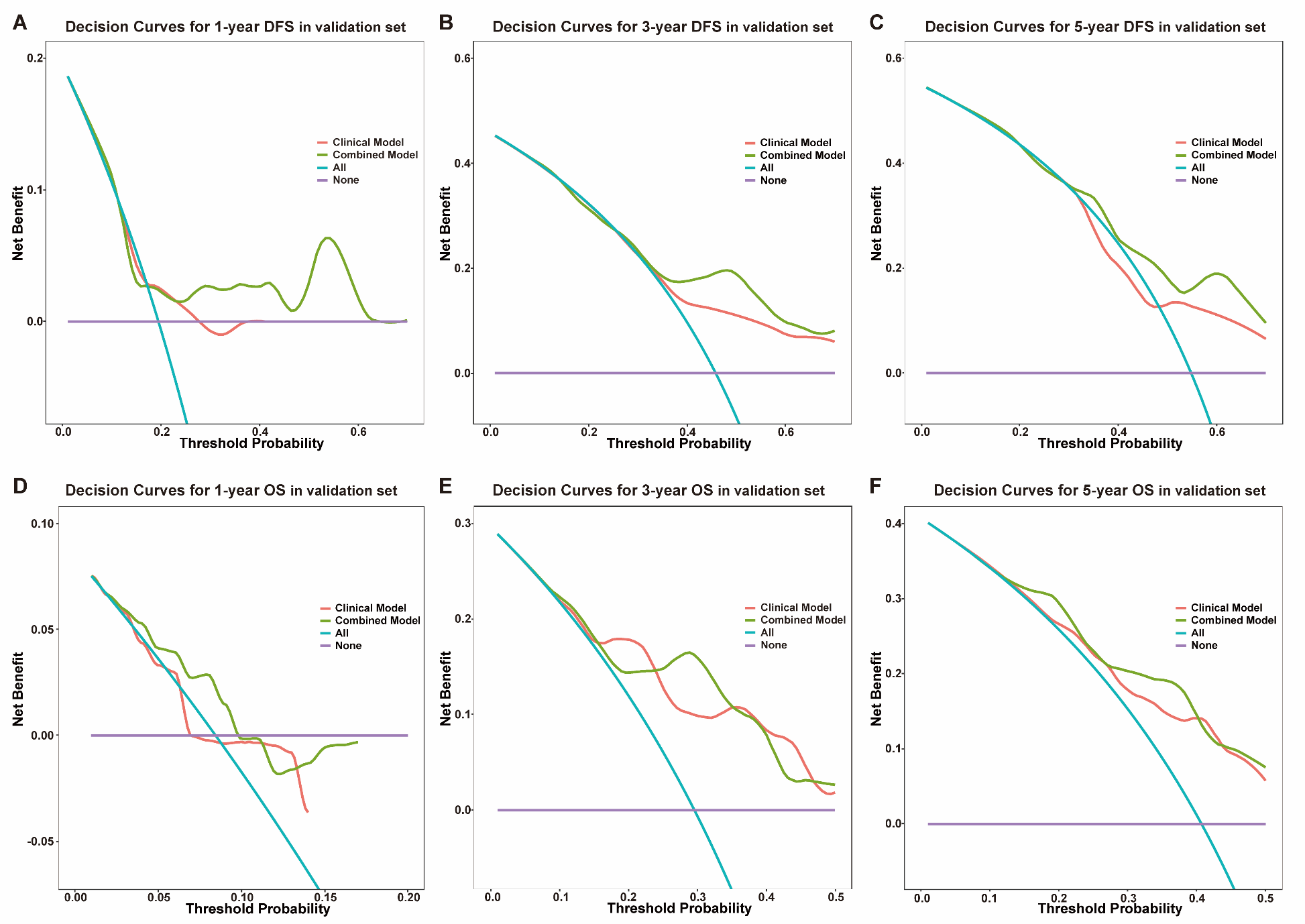


**Figure S4** The DCA curves of the models in validation cohorts. **A-C**, The DCA curves of the comparison between the clinical model and combined model to predict the 1-, 3-, and 5-year DFS. **D-F**, The DCA curves of the comparison between clinical model and combined model to predict the 1-, 3-, and 5-year OS. DCA curves showed that the model with Rad-score benefits for patients in the prediction of DFS and OS at 1, 3, and 5 years.
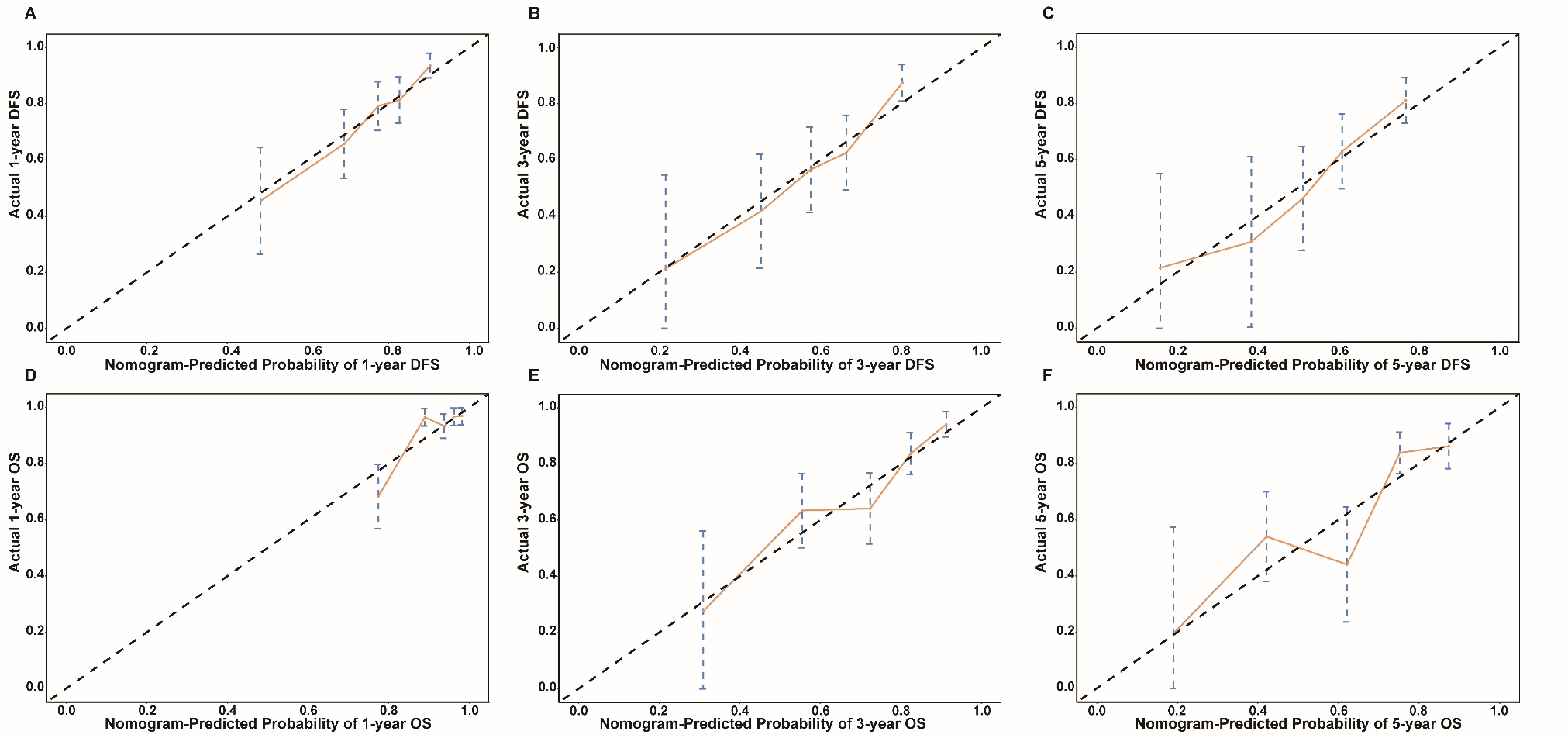


**Figure S5** To predict the DFS and OS of NSCLC using the nomogram and calibrate for the predictive model. The diagonal dotted line represents the ideal state, and the solid red line represents the actual predictive value: the closer it is to the diagonal dotted line, the better the predictive power

**Table S1** Definition and Biological Relevance of Radiomic Features in Rad-Score

| **Radiomic Feature** | **Category** | **Definition** | **​​Biological Basis** |
| --- | --- | --- | --- |
| wavelet-LHL_glszm_SmallAreaHighGrayLevelEmphasis | Texture | Quantifies the emphasis on small-area, high-intensity homogeneous connected regions within the Gray Level Size Zone Matrix (GLSZM) computed from the LHL wavelet-transformed image. | Accentuates the distribution of spatially compact, hyperintense regions, potentially associated with the presence of dense cellular clusters, microcalcifications, foci of microvascular proliferation, or other hyperdense/hypermetabolic microlesions within tumor tissue. |
| wavelet-LLH_glszm_ZoneVariance | Texture | Quantifies the variance of voxel intensity values within all homogeneous zones of the Gray Level Size Zone Matrix (GLSZM) computed from LLH wavelet-transformed images. | Characterizes the dispersion of intensity values within homogeneous zones, potentially associated with heterogeneity in tumor microarchitecture, admixed tissue constituents, or subtle textural variations along the axial dimension. |
| wavelet-HHL_gldm_DependenceVariance | Texture | Quantifies the variance in the distribution of gray-level dependence counts within the Gray Level Dependence Matrix (GLDM) computed from HHL wavelet-transformed images. | Characterizes the spatial consistency of regional textural patterns, potentially associated with underlying tumor heterogeneity, microstructural complexity, or invasive potential. |
| wavelet-HLL_gldm_LowGrayLevelEmphasis | Texture | quantifies the weighting/emphasis toward low-gray-level voxels and their spatial dependencies within the Gray Level Dependence Matrix (GLDM) computed from HLL wavelet-transformed images. | Exhibits sensitivity to the spatial distribution of hypointense regions, potentially associated with the presence of necrotic areas, cystic components, edema, or low-cellularity regions within tumor tissue. |

**Table S2** C-index was used to evaluate the predictive performance of the clinical and combined models for DFS and OS

|  | Training Set(N=166) | Validation Set(N=72) |
| --- | --- | --- |
| DFS |  |  |
| Clinical model | 0.655(0.599-0.712) | 0.635(0.546-0.724) |
| Combined model | 0.704(0.649-0.760) | 0.674(0.578-0.770) |
| OS |  |  |
| Clinical model | 0.684(0.619-0.749) | 0.664(0.572-0.757) |
| Combined model | 0.748(0.691-0.806) | 0.693(0.603-0.783) |

Abbreviations: DFS, disease-free survival; OS, overall survival
